# Sleep Deprivation Triggers the Excessive Activation of Ovarian Primordial Follicles via β2 Adrenergic Receptor Signaling

**DOI:** 10.1002/advs.202402393

**Published:** 2024-09-04

**Authors:** Lichun Weng, Hanqing Hong, Qinyu Zhang, Chengqi Xiao, Qiuwan Zhang, Qian Wang, Ju Huang, Dongmei Lai

**Affiliations:** ^1^ The International Peace Maternity and Child Health Hospital School of Medicine Shanghai Jiao Tong University Shanghai 200030 China; ^2^ Shanghai Key Laboratory of Embryo Original Diseases Shanghai 200030 China; ^3^ Songjiang Hospital and Songjiang Research Institute Shanghai Key Laboratory of Emotions and Affective Disorders Shanghai Jiao Tong University School of Medicine Shanghai 201600 China

**Keywords:** β2 adrenergic receptor, KIT ligand, ovarian function, primordial follicles, sleep deprivation

## Abstract

Sleep deprivation (SD) is observed to adversely affect the reproductive health of women. However, its precise physiological mechanisms remain largely elusive. In this study, using a mouse model of SD, it is demonstrated that SD induces the depletion of ovarian primordial follicles, a phenomenon not attributed to immune‐mediated attacks or sympathetic nervous system activation. Rather, the excessive secretion of stress hormones, namely norepinephrine (NE) and epinephrine (E), by overactive adrenal glands, has emerged as a key mediator. The communication pathway mediated by the KIT ligand (KITL)‐KIT between granulosa cells and oocytes plays a pivotal role in primordial follicle activation. SD heightened the levels of NE/E that stimulates the activation of the KITL‐KIT/PI3K and mTOR signaling cascade in an β2 adrenergic receptor (ADRB2)‐dependent manner, thereby promoting primordial follicle activation and consequent primordial follicle loss in vivo. In vitro experiments further corroborate these observations, revealing that ADRB2 upregulates KITL expression in granulosa cells via the activation of the downstream cAMP/PKA pathway. Together, these results reveal the significant involvement of ADRB2 signaling in the depletion of ovarian primordial follicles under sleep‐deprived conditions. Additionally, ADRB2 antagonists are proposed for the treatment or prevention of excessive activation of primordial follicles induced by SD.

## Graphical abstract

Sleep deprivation induces adrenal hyperactivity, leading to increased secretion of norepinephrine and epinephrine, which activate ADRB2 receptors in the ovaries. This activation triggers the overexpression of KIT/KITL through the ADRB2/cAMP/PKA pathway, excessively activating primordial follicles and reducing ovarian reserve. ADRB2 antagonists may mitigate these adverse effects on ovarian health, suggesting a potential treatment for sleep‐deprivation‐induced primordial follicle activation.

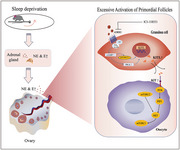

## 1 Introduction

Sleep plays a vital role in health and well‐being, affecting not only cognitive functioning, mood, mental health, cardiovascular, cerebrovascular, and metabolic health,^[^ ^1^ ^]^ but also ovarian function in women.^[^ ^2^ ^]^ A prospective cohort study revealed a positive association between extended sleep duration and an increased chance of successful embryo transfer.^[^ ^3^ ^]^ The primary reason for not completing embryo transfer often revolves around an inadequate ovarian response to stimulation, resulting in cancellation prior to egg retrieval.^[^ ^4^ ^]^ This suggests that a decrease in sleep duration could affect ovarian function. In another prospective cohort study that examined the correlation between sleep monitoring using wearable devices and assisted reproductive therapy, individuals experiencing recurrent implantation failure exhibited a notable decrease in their average daily sleep duration compared to the control group, with no significant variance in sleep quality,^[^ ^5^ ^]^ indicating that shortened sleep duration could potentially serve as a risk factor for recurrent implantation failure. Previous research has also indicated that disruptions to the biological clock can affect various aspects of reproductive physiology, including the menstrual cycle and sex hormone levels,^[^ ^6^ ^]^ and even interfere with ovulation in the ovaries.^[^ ^7^ ^]^ One systematic review further demonstrated that female fertility and IVF outcomes may be affected by short sleep duration, evening chronotypes, or shift/night work schedules.^[^ ^2^ ^]^ We recently reported that long‐term negative event related chronic stress and sleep problems exist in patients with idiopathic premature ovarian insufficiency/failure (POI/POF) prior to diagnosis.^[^ ^8^ ^]^ However, the molecular mechanisms underlying this association have not yet been fully elucidated.

Normal ovarian function maintains both the female reproductive lifespan and healthspan. Premature ovarian aging reduces fertility and overall health quality, and leads to premature mortality.^[^ ^9^ ^]^ Within the ovary, there is a pool of follicles at various stages, of which the primordial follicles constitute the ovarian reserve, and their numbers are finite at birth. As women age, the number of primordial follicles decreases, and ovarian aging ultimately occurs.^[^ ^10^ ^]^ Preserving the majority of primordial follicles in a dormant state over an extended period is essential to safeguard the initial follicle reserve. Heightened activation of dormant primordial follicles and accelerated follicular atresia are the primary causes of premature depletion of the ovarian reserve, leading to premature ovarian aging.^[^ ^11^ ^]^ KIT ligand (KITL), also known as stem cell factor (SCF), is a ligand for the KIT receptor, a receptor tyrosine kinase (RTK). Upon binding of the receptor and ligand, various signaling pathways, including the phosphoinositide 3‐kinase (PI3K)/AKT and JAK/STAT pathways, are activated.^[^ ^12^ ^]^ This involvement in signaling pathways contributes to the regulation of cell proliferation, differentiation, survival, and migration. Although KITL is not essential for primordial follicle formation, it is crucial for the further development of primary follicles.^[^ ^13^ ^]^ Primordial follicle granulosa cells (pfGCs) interact with oocyte KIT through KITL, activating the PI3K signaling cascade, and triggering the awakening of oocyte cells.^[^ ^14^ ^]^ Our previous studies reported that chronic unpredictable stress leads to excessive activation of primordial follicles in mouse ovaries, increases oxidative stress damage in the ovaries, and accelerates ovarian aging.^[^ ^15^ ^]^ However, the underlying mechanisms remain unclear.

In this study, we established a sleep deprivation (SD) female mouse model based on previous studies^[^ ^16^ ^]^ to uncover the mechanisms by which SD exerts lasting effects on ovarian function. We showed that SD strongly results in the loss of ovarian primordial follicles through overactive adrenal glands, which produce excessive amounts of the stress hormones, norepinephrine (NE) and epinephrine (E). We further show that SD‐induced elevation of NE/E level, in an β2 adrenergic receptor (ADRB2) ‐dependent manner, induces the activation of KITL‐KIT/PI3K and mTOR pathways involved in follicle activation, leading to the loss of primordial follicles in vivo. In vitro, ADRB2 activates the downstream kinase cAMP/PKA (Protein Kinase A), thereby upregulating KITL expression in granulosa cells. We propose that SD triggers the activation of ovarian primordial follicles through the ADRB2 signaling pathway, which may play a role in POI/POF. A better understanding of the molecular basis of SD‐induced premature ovarian aging will ultimately lead to the development of preventive measures against such complex diseases.

## 2 Results

### 2.1 SD Leads to the Loss of Primordial Follicles

**Figure 1:**
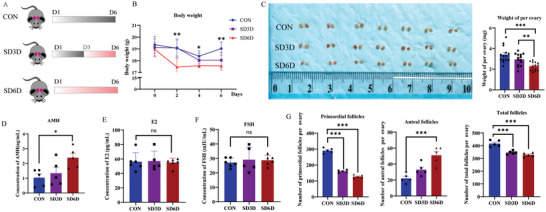
SD induces the loss of primordial follicles. A) Experimental design diagram of three groups of 6–8 week‐old female mice subjected to SD experiments, with mice deprived of sleep for 0, 3, and 6 days, respectively. B) Significant decrease in body weight of mice after SD over time (CON *n* = 6; SD3D *n* = 6; SD6D *n* = 6). Statistical method: One‐way ANOVA with LSD Test. C) Representative images of mouse ovaries after SD treatment, showing a significant reduction in ovarian weight in sleep‐deprived mice (CON *n* = 14; SD3D *n* = 14; SD6D *n* = 14). Statistical method: One‐way ANOVA with LSD Test. D) The serum AMH levels in mice significantly increased after 6 days of sleep deprivation (CON *n* = 5; SD3D *n* = 5; SD6D *n* = 5). Statistical method: One‐way ANOVA with LSD Test. E‐F) There were no significant differences in serum E2 and FSH levels after sleep deprivation (CON *n* = 6; SD3D *n* = 5; SD6D *n* = 6). Statistical method: One‐way ANOVA. G) Number of primordial follicles, antral follicles, total follicles count in mice after SD treatment (CON *n* = 5; SD3D *n* = 5; SD6D *n* = 5). Statistical methods: One‐way ANOVA with LSD Test. Data are presented as mean with SEM. *p < 0.05, **p < 0.01, ***p < 0.001, and ns indicates not significant. CON, control; SD, sleep deprivation.

To determine the effect of SD on ovarian function, we conducted a study involving mice subjected to 0, 3, and 6 days of SD, as illustrated in **Figure** **1**A. The body weights of the mice significantly decreased following the period of SD (Figure 1B). Subsequently, upon weighing the mice's ovaries, a considerable reduction in ovarian weight was observed after 6 days of SD treatment (Figure 1C; Figure S1A, Supporting Information). Serum AMH levels in mice significantly increased after six days of SD (Figure 1D). However, there were no considerable differences in the serum E2 and FSH levels after SD (Figure 1E,F). We attempted to further observe the impact of SD on mouse ovarian follicles. Subsequently, following tissue sectioning and Hematoxylin and Eosin (HE) staining (Figure S1D, Supporting Information), the ovarian follicles of the mice were examined, which showed that there was a substantial reduction in both the number of primordial follicles and total follicles, and a considerable increase in the number of antral follicles (Figure 1G), indicating that the ovaries are highly sensitive to SD, leading to the loss of primordial follicles.

We also conducted experiments using another SD model, the water platforms (Figure S2A, Supporting Information). Mice were placed on water platforms, where they could move between columns and had access to drinking water and ample food. When muscle tone was lost during sleep, they would touch the water and wake up. In this setup, we limited SD experiment to 0 and 1 day. Detailed experimental procedures are provided in the Supporting Information. After 1 day of sleep deprivation, mice showed significant reductions in body weight, ovarian weight, and the ratio of ovarian weight to body weight (Figure S2B–E, Supporting Information). Subsequently, we measured serum levels of NE, E, and AMH in mice after 0 and 1 day of sleep deprivation. The levels of NE, E, and AMH were significantly elevated in the serum of sleep‐deprived mice (Figure S2F–H, Supporting Information). The analysis of follicle counting of mice ovaries indicated that sleep deprivation induced the loss of primordial follicles (Figure S2I, Supporting Information). These findings suggest that the water platform‐induced SD model also causes the loss of primordial follicles.

### 2.2 Single‐Cell RNA‐Seq Profiling of Ovarian Cells after SD and the Role of KIT in Activating Ovarian Primordial Follicles

Initially, we conducted RNA‐Seq on ovarian tissues from mice subjected to 0 and 6 days of SD. RNA‐seq data demonstrated changes in gene expression, and Gene Ontology (GO) analysis revealed several biological processes involved in oocyte maturation, oogenesis, ossification, regulation of system processes, and osteoblast differentiation (Figure S3B,D, Supporting Information). KITL was identified as one of the top 25 differential expressed genes (DEGs) (Figure S3C, Supporting Information), and it was found to be the gene most closely related to follicle development within this group.

**Figure 2:**
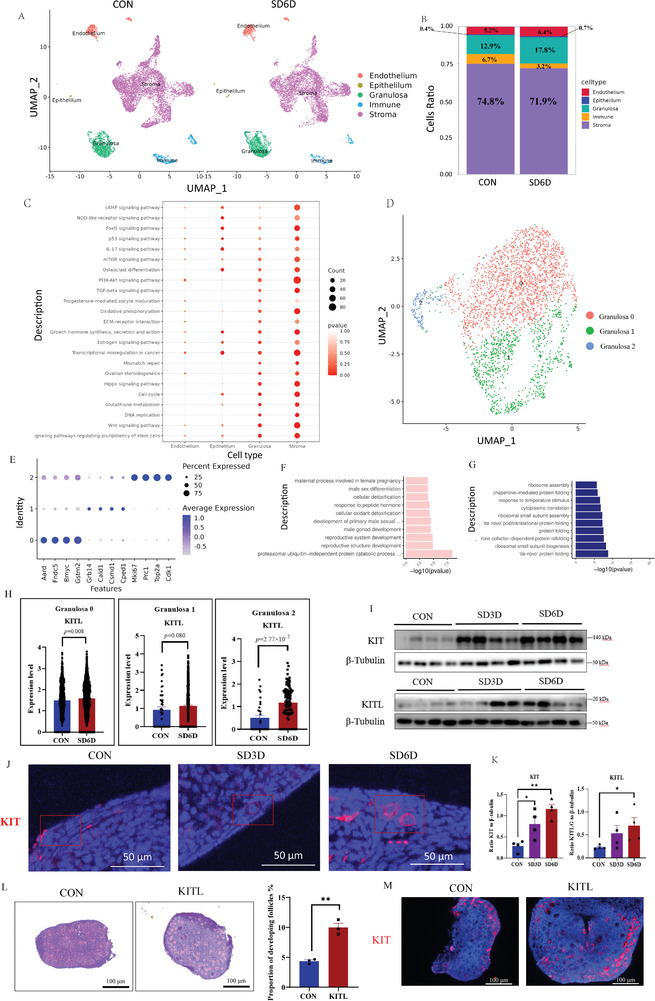
Single‐cell transcriptome profiling of mouse ovarian cells after SD and the role of KIT in activating ovarian primordial follicle. A) Uniform Manifold Approximation and Projection (UMAP) plot of different cell clusters in mouse ovaries. B) The proportion of stromal cells, granulosa cells, endothelial cells, immune cells, and epithelial cells in mouse ovarian cells after 0 and 6 days of SD. C) Representative KEGG enrichment pathways for endothelial cells, epithelial cells, granulosa cells, and stromal cells. D) UMAP plot of granulosa cells showing three subpopulations: granulosa cells 0, 1, and 2. E) Expression markers of the three granulosa cell subpopulations. F) Significantly upregulated differentially expressed genes (DEGs) enriched GO terms (biological processes) of DEGs in granulosa cell cluster 0. G) Significantly downregulated enriched GO terms (biological processes) of DEGs in granulosa cell cluster 0. H) Expression of KITL in granulosa cells across different clusters. I) Western Blot (WB) analysis revealing the high expression of KIT/KITL in ovarian tissue after SD. J) Immunofluorescence analysis of ovarian tissue showing overexpression of KIT in primordial follicles after SD. K) Expression analysis graph of KIT/KITL in ovarian tissue after SD via WB Experiment (CON *n* = 4; SD3D *n* = 4; SD6D *n* = 4). Statistical method: One‐way ANOVA with LSD Test. L) HE staining and proportion analysis of developing follicles in ovarian tissue of 6 days old mice after adding KITL (500 ng mL^−1^) to the culture medium for 4 days. Scale bar, 100 µm. (CON *n* = 3; KITL *n* = 3), Statistical method: Independent Samples Test. M) Immunofluorescence analysis showing increased expression of KIT in oocytes of ovarian tissue after adding KITL to the culture medium. Scale bar, 100 µm. Data are presented as mean with SEM. *p < 0.05, and **p < 0.01. CON, control; SD, sleep deprivation; KITL, KIT ligand.

To further explore the effect of SD on different types of ovarian cells, we performed 10X single‐cell RNA‐seq. Using a Uniform Manifold Approximation and Projection (UMAP) algorithm for nonlinear dimensionality reduction (**Figure** **2**A), we identified various cell types based on gene clustering analyses and previous studies.^[^ ^17^ ^]^ Using this sequencing method for ovarian tissue often results in low or negligible capture of oocytes (Figure S4, Supporting Information). The cells were categorized as stromal, granulosa, endothelial, immune, and epithelial cells (Figure 2B). KEGG pathway enrichment analysis of these cell types indicated that SD affects pathways related to ovarian and follicle development, including the Wnt signaling, Hippo signaling, ovarian steroidogenesis, estrogen signaling, TGF‐beta signaling, PI3K‐AKT signaling, osteoclast differentiation, mTOR signaling, FoxO signaling, and cAMP signaling pathway (Figure 2C).

The UMAP plot of the granulosa cell cluster revealed three subpopulations: granulosa 0, granulosa 1, and granulosa 2 (Figure 2D). The expression markers of these subclusters are shown in Figure 2E. On the basis of previous study,^[^ ^17a^ ^]^ Granulosa 1 is likely associated with atretic follicles. Granulosa 2 exhibits differential expression of genes related to cell proliferation and meiosis, which José V. V. Isola et al. termed mitotic granulosa cells.^[^ ^17b^ ^]^ This subpopulation represents a smaller fraction of granulosa cells. Granulosa 0 comprised the largest group, accounting for over 60% of all granulosa cells. To investigate the biological impact of SD on granulosa 0, we conducted GO analysis. The analysis indicated that sleep deprivation affected various biological processes in granulosa cells, including reproductive structure development, reproductive system development, gonad development, male sex differentiation, and maternal processes involved in female pregnancy (Figure 2F). Additionally, processes such as protein folding, cellular transport, ribosome biogenesis, and tissue development were also affected (Figure 2G).

Expression analysis of KIT across the three granulosa subpopulations revealed significantly upregulated levels in granulosa cells 0 and 2 (Figure 2H). Western blot analysis of ovarian tissue further confirmed a considerable increase in the expression levels of KIT and KITL in mice after SD (Figure 2I,K). Immunofluorescence staining of the ovaries indicated that KIT proteins were localized in primordial follicles (Figure 2J). The KIT‐KITL signaling pathway plays a crucial role in communication between granulosa cells and oocytes, triggering follicle activation via the PI3K signaling cascade, essential for follicle development, proliferation, and survival.^[^ ^14^, ^18^ ^]^ To further investigate the role of KITL in follicle activation, we conducted ex vivo cultures of mouse ovaries. After culturing ovarian tissues from 6‐day‐old mice ex vivo for 4 days, we observed significant follicle activation and an increase in the proportion of developing follicles in tissues treated with KITL (Figure 2L). Moreover, KIT expression was elevated in KITL‐treated oocytes (Figure 2M).

### 2.3 The activation of Primordial Follicles Induced by SD

Nest, we explored the possible mechanism underlying primordial follicle loss induced by SD (Figure S5A, Supporting Information). The currently identified causes associated with the loss of primordial follicles include apoptosis of primordial follicles, ferroptosis, and excessive activation.^[^ ^19^ ^]^ Either positive apoptosis signal or positive ferroptosis signal was detected in primordial follicles of ovaries in SD mice. However, a positive TUNEL signal was observed in the antral follicles of the ovaries of SD mice (Figure S5B, Supporting Information). Similarly, positive Prussian blue staining was observed in antral follicles and ovarian stroma (Figure S5C, Supporting Information). Thus, the apoptosis and ferroptosis do not the directly damage the primordial follicles.

**Figure 3:**
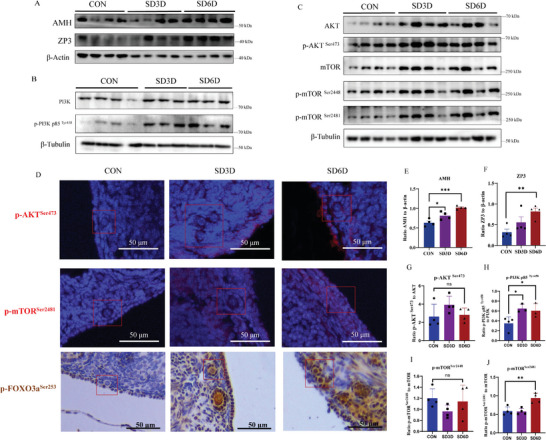
A) The protein expression of AMH and ZP3 increase in mice ovaries after SD by Western blots. B, C) Western blots analysis of the activation of phosphorylated PI3K/AKT and mTOR pathways in ovarian tissue. D) Immunostaining analysis of ovarian tissue showing overexpression of p‐AKT^Ser473^, p‐mTOR^Ser2481^, and p‐FOXO3a^Ser253^ in primordial follicles after SD. E–J) Protein expression analysis chart of AMH, ZP3, phosphorylated PI3K/AKT, and mTOR (E–G, I, J: CON *n* = 4; SD3D *n* = 4; SD6D *n* = 4. H: CON *n* = 4; SD3D *n* = 3; SD6D *n* = 3). Statistical method: E, H–J: One‐way ANOVA with LSD Test; F, G: Independent‐Samples Kruskal‐Wallis Test. Data are presented as mean with SEM. *p < 0.05, **p < 0.01, and ns indicates not significant. CON, control; SD, sleep deprivation.

The activation of primordial follicles leads to further growth and development of follicles, ultimately resulting in ovulation or atresia. Excessive activation of primordial follicles during this process can lead to the premature depletion of the follicle pool.^[^ ^19^, ^20^ ^]^Anti‐Müllerian hormone (AMH) is mainly produced by small developing antral follicles,^[^ ^21^ ^]^ we observed a considerable increase in serum AMH concentration in sleep‐deprived mice (Figure 1D), and Western blots revealed a marked upregulation of AMH and Zona pellucida sperm‐binding protein 3 (ZP3) proteins in ovarian tissues (**Figure** **3**A,E,F), consistent with the increased count of ovarian antral follicles in SD mice (Figure 1E), suggesting a potential overactivation of primordial follicles during SD.

The PI3K and mTOR pathways activate dormant primordial follicles, prompting further development and growth of the primordial follicles.^[^ ^14^ ^]^ Western blotting further confirmed the phosphorylation levels of key pathways, PI3K and mTOR, involved in the activation of ovarian follicles after SD (Figure 3C,H–J). The p‐AKT/AKT ratio did not show a statistically significant difference (Figure 3B,G), while p‐AKT relative to β‐Tubulin significantly increased. This suggests that SD may have led to an increase in total AKT expression. Immunofluorescence staining of the ovaries revealed the localization of these proteins in the primordial follicles (Figure 3D). These results further indicate that a potential overactivation of primordial follicles during SD in mice ovaries.

### 2.4 The Loss of Primordial Follicles Induced by SD Is Dependent on the Functionality of the Adrenal Glands, but Not on the Activation of Sympathetic Nerves or an Immune Attack

In order to define how the effects of SD were transmitted to the ovaries and decreased the number of primordial follicles, several underlying cause, including neural activation, immune factors, and adrenal activation, were examined (Figure S6A¸ Supporting Information). RNA sequencing of mouse ovaries subjected to 0 and 6 days of SD revealed a significant increase in the expression of the sympathetic nerve marker tyrosine hydroxylase (TH) in the ovaries of SD mice (Figure S6B, Supporting Information). Previous clinical evidence has shown the activation of the sympathetic nervous system (SNS) in patients with sleep disorders and SD.^[^ ^22^ ^]^ Therefore, we further conducted an experiment in which mice with 6‐hydroxydopamine (6‐OHDA) to mimic sympathetic chemical ablation. Subsequently, they were exposed to a 6‐day period of SD to explore whether ablation of the sympathetic nervous system could alleviate the loss of primordial follicles. We validated the model using TH immunofluorescence (Figure S6D, Supporting Information). We observed that the ovarian weight of the mice did not recover following treatment with either 2 or 200 mg kg^−1^ 6‐OHDA (Figure S6C,E, Supporting Information). The number of primordial and overall follicles significantly decreased, and the number of antral follicles significantly increased in mice after SD (Figure S4F, Supporting Information). Treatment with either 2 or 200 mg kg^−1^ 6‐OHDA did not reverse the ovarian damage caused by SD (Figure S6E,F, Supporting Information). Collectively, these data indicate that excessive activation of the sympathetic nervous system is not the main cause of reduction in primordial follicles in SD mice.

Sleep plays a dynamic role in modulating the immune system by affecting the physiological processes that affect the circulation of immune cells and the secretion of inflammatory cytokines.^[^ ^23^ ^]^ Immunological abnormalities are a category of etiological factors in POI, often involving immune‐mediated inflammation that selectively affects ovarian follicles.^[^ ^24^ ^]^ In mice experiments, it has been found that the progressive decline in ovarian function is demonstrated to be due to the activation of CD4 + T cells and infiltration of ovarian lymphocytes.^[^ ^25^ ^]^ Therefore, recombination‐activating gene 1 knockout (*Rag1* KO) mice were used to explore whether the reduction in primordial follicles in the ovaries was caused by SD induced immune attacks. In *Rag1* KO mice, the early development of T and B lymphocytes was severely blocked, preventing the generation of mature T and B lymphocytes.^[^ ^26^ ^]^ However, *Rag1* KO mice can reproduce normally during their reproductive age. It is noteworthy that the spleen CD3 expression in *Rag1* KO mice was negative (Figure S7A, Supporting Information). After *Rag1* KO mice underwent 6 days of SD, the number of primordial follicles and total follicles in *Rag1* KO mice still showed a substantial decrease (Figure S7C, Supporting Information), indicating that the reduction in the number of primordial follicles in mice subjected to SD was not primarily driven by an immune attack. Thus, the significant decrease in primordial follicle count cannot be attributed to either sympathetic nerve activation or immune attacks.

**Figure 4:**
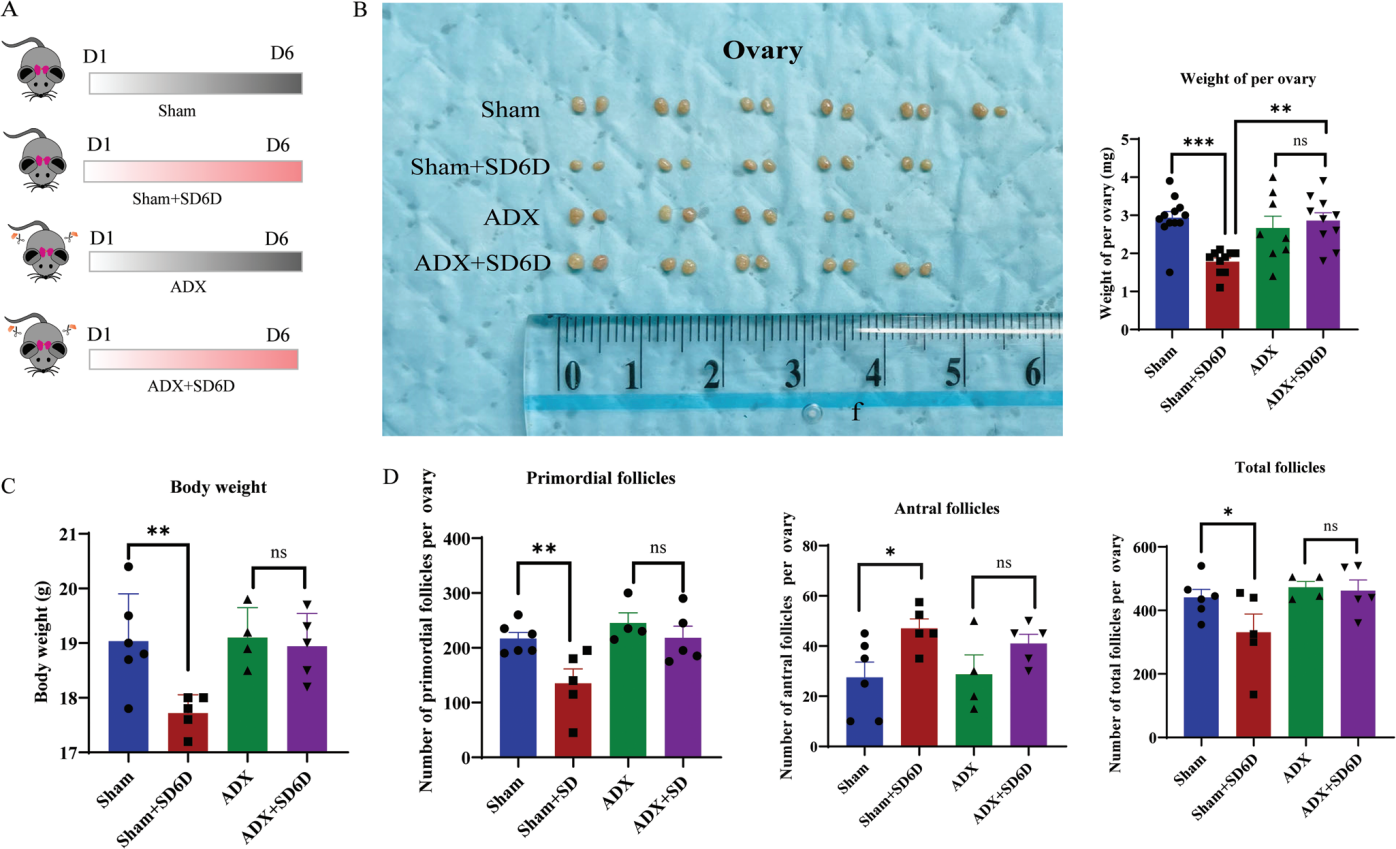
The loss of primordial follicles induced by SD is dependent on the adrenal glands. A) Experimental design for SD in sham‐operated mice and ADX mice. B) Representative images of ovaries and changes in ovarian weight after experimentation in four groups: Sham, Sham + SD6D, ADX, and ADX + SD6D mice (Sham, *n* = 12; Sham + SD6D *n* = 10; ADX *n* = 8; ADX + SD6D *n* = 10). Statistical method: One‐way ANOVA with Tamhane's T2 Test. C) Changes in body weight after experimentation in the four groups of mice, showing a significant increase in body weight in SD mice after adrenal gland removal compared to Sham + SD6D mice (Sham, *n* = 6; Sham + SD6D *n* = 5; ADX *n* = 4; ADX + SD6D *n* = 5). Statistical method: One‐way ANOVA with LSD Test. D) Number of primordial follicles, antral follicles and total follicle count after experimentation in the four groups of mice (Sham, *n* = 6; Sham + SD6D *n* = 5; ADX *n* = 4; ADX + SD6D *n* = 5). Statistical method: One‐way ANOVA with LSD Test. Data are presented as mean with SEM. *p < 0.05, **p < 0.01, ***p < 0.001, and ns indicates not significant. SD, sleep deprivation; ADX, adrenalectomy.

SD, like stress, enhances adrenal gland reactivity in healthy adults.^[^ ^27^ ^]^ Adrenalectomy (ADX) was performed in mice to investigate the contribution of the glands (**Figure** **4**A). Remarkably, no substantial variance was observed in ovarian weight, body weight, primordial follicle count, or total follicle number between mice undergoing adrenal resection with or without SD (Figure 4B–D). In addition, the underexpression of immunostaining of KIT, p‐AKT^Ser473^, and p‐FOXO3a^Ser253^ in primordial follicles in ADX + SD6D group were observed compared with Sham + SD6D group (Figure S8B, Supporting Information). Hence, the adrenal gland may be a crucial factor in orchestrating the noteworthy decrease in ovarian weight, primordial follicle count, and total follicle number in mice following SD.

### 2.5 Both Noradrenaline (NE) and Epinephrine (E) Contribute to the Loss of Primordial Follicles

**Figure 5:**
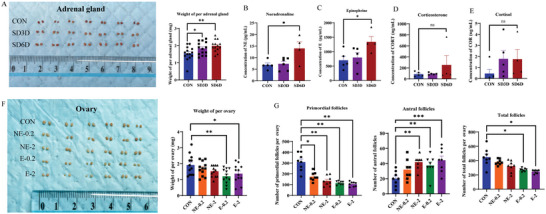
Both noradrenaline (NE) and epinephrine (E) induce the loss of primordial follicles. A) Adrenal gland weight significantly increased in mice after 0, 3, and 6 days of SD (CON *n* = 14; SD3D *n* = 14; SD6D *n* = 14). Statistical method: One‐way ANOVA with LSD Test. B‐E) Levels of stress hormones NE, E, CORT, and COR in mice after 0, 3, and 6 days of SD (NE: CON *n* = 5; SD3D *n* = 6; SD6D *n* = 4) (E, CORT, and COR: CON *n* = 5; SD3D *n* = 5; SD6D *n* = 4). Statistical methods: NE, E, One‐way ANOVA with LSD Test; CORT, COR, Nonparametric tests. F) Representative images of ovaries and changes in ovarian weight in mice after treatment with vehicle, NE (0.2 and 2 mg kg^−1^, intraperitoneal injections for 6 days), and E (0.2 and 2 mg kg^−1^ intraperitoneal injections for 6 days) (CON *n* = 12, NE‐0.2 *n* = 12, NE‐2 *n* = 12, E‐0.2 *n* = 12, E‐2 *n* = 12). Statistical method: One‐way ANOVA with LSD Test. G) Number of primordial follicles, antral follicles and total follicle count in mice after each experimentation (CON n = 8, NE‐0.2 *n* = 8, NE‐2 *n* = 8, E‐0.2 *n* = 8, E‐2 *n* = 8). Statistical method: Primordial follicles: One‐way ANOVA with Tamhane's T2 Test; Antral follicles: One‐way ANOVA with LSD Test; Total follicles: Independent‐Samples Kruskal‐Wallis Test. Data are presented as mean with SEM. In all panels, *p < 0.05, **p < 0.01, ***p < 0.001, and ns indicates not significant. CON, control; SD, sleep deprivation; NE, noradrenaline; E, epinephrine; CORT, corticosterone; COR, Cortisol.

In the present study, we collected and analyzed the adrenal glands of mice subjected to SD for 0, 3, and 6 days. After 3 and 6 days of SD, the adrenal gland weight of the mice significantly increased (**Figure** **5**A). This finding indicates that SD results in adrenal hyperactivity. We then analyzed the stress hormone levels in the serum of mice and observed a considerable increase in NE (Figure 5B) and E (Figure 5C) after SD. However, there were no statistically significant differences in corticosterone (CORT) (Figure 5D) and cortisol (COR) concentrations (Figure 5E).

Subsequently, we explored whether the ovaries of the mice exhibited similar responses by treating them with different concentrations of NE and E. We measured the peripheral NE or E levels in mice after injection of NE or E to validate the success of the model (Figure S9A,B, Supporting Information). After administering doses of 0.2 and 2 mg kg^−1^ of E to mice, we observed a substantial reduction in ovarian weight (Figure 5F). Although there was no significant difference in ovarian weight after treatment with NE, it exhibited a decreasing trend (Figure 5F). Intriguingly, both NE and E treatments reduced the number of primordial follicles in mouse ovaries. Treatment with E also considerably decreased the total number of ovarian follicles. Both NE and E led to a loss of ovarian primordial follicles in mice, whereas the number of antral follicles increased (Figure 5G). These results indicated that the adrenergic hormones E and NE directly decreased the number of primordial follicles in mouse ovaries.

### 2.6 The Activation of ADRB2 Induces the Loss of Primordial Follicles

Stress hormones, both NE and E, have been shown to commonly trigger downstream signaling pathway via adrenergic receptors, which are G‐protein coupled receptors (GPCRs).^[^ ^28^ ^]^ Various types of adrenergic receptors in granulosa cells were analyzed using single‐cell RNA‐seq, and the results showed that only the ADRB2 receptor was significantly upregulated after SD (Figure S10D–K, Supporting Information). Immunofluorescence analysis revealed a strong positive expression of ADRB2 in granulosa cells within the ovarian tissues of mice after SD (Figure S10L, Supporting Information). Treatment with the ADRB2 receptor agonists clenbuterol (CLEN) and salbutamol (SAL) led to the upregulation of ADRB2 receptor expression in the granulosa cell of ovarian tissue (Figure S10M,N, Supporting Information). Therefore, we focused on targeting the ADRB2 receptor.

**Figure 6:**
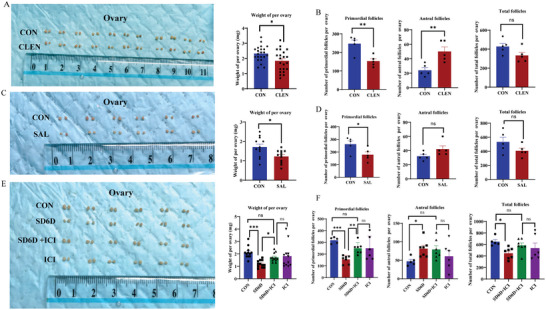
Activation of the ADRB2 signaling pathway induces the loss of primordial follicles. A) Significant reduction in ovarian weight in mice after administering water containing ADRB2 agonist CLEN for 6 days (CON *n* = 22, CLEN *n* = 22). Statistical method: Mann‐Whitney test. B) Number of primordial follicles, antral follicles, and total follicle count in mice after treatment with ADRB2 agonist CLEN (CON *n* = 5, CLEN *n* = 5). Statistical method: Independent Samples Test. C) Significant reduction in ovarian weight in mice after intraperitoneal administration of another ADRB2 agonist SAL for 6 days (CON *n* = 12, SAL *n* = 12). Statistical method: Independent Samples Test. D) Number of primordial follicles, antral follicles and total follicle count in mice after treatment with ADRB2 agonist SAL (CON *n* = 5, SAL *n* = 5). Statistical method: Independent Samples Test. E) ADRB2 antagonist ICI was intraperitoneally injected to rescue SD mice, showing significant recovery of ovarian weight in mice after addition of ICI to SD6D mice (CON *n* = 12, SD6D *n* = 12, SD6D + ICI *n* = 12, ICI *n* = 12). Statistical method: Independent‐Samples Kruskal‐Wallis Test. F) Number of primordial follicles, antral follicles and total follicle count in mice after treatment with ADRB2 antagonist ICI (CON *n* = 5, SD6D *n* = 5, SD6D + ICI *n* = 5, ICI *n* = 5). Statistical methods: Primordial follicles: Independent‐Samples Kruskal‐Wallis Test; Antral follicles: One‐way ANOVA with LSD Test; Total follicles: Independent‐Samples Kruskal‐Wallis Test. Data are presented as mean with SEM. *p < 0.05, **p < 0.01, ***p < 0.001, and ns indicates not significant. CON, control; SD, sleep deprivation; CLEN, Clenbuterol; SAL, Salbutamol; ICI, ICI‐118551.

Next, we investigated whether ADRB2 activation affects primordial follicles using an ADRB2 receptor agonist (CLEN and SAL)‐treated mouse model. After administering water containing CLEN to mice for 6 days, the ovarian weight (**Figure** **6**A) and number of primordial follicles decreased significantly (Figure 6B), while the number of antral follicles increased significantly (Figure 6B). Similarly, 6 days after the intraperitoneal injection of SAL, both ovarian weight (Figure 6C) and the number of primordial follicles (Figure 6D) significantly decreased, along with a declining trend in the total follicle count. The number of antral follicles showed an increasing trend, however, this was not statistically significant (Figure 6D).

To further substantiate the involvement of the ADRB2 signaling pathway in the activation of primordial follicles during SD, sleep‐deprived mice were intraperitoneally injected with the ADRB2 antagonist ICI‐118551 (ICI) to assess its potential rescuing effect. The experimental groups were treated with a daily injection of DMSO, DMSO in conjunction with SD, ICI, and ICI in conjunction with SD. When comparing these results to SD mice injected with DMSO, it was evident that the ovarian weight of SD mice following ICI treatment exhibited a substantial increase (Figure 6E). Furthermore, the number of primordial follicles, antral follicles, and total follicles (Figure 6F) in sleep‐deprived mice after ICI treatment did not display substantial differences compared to mice injected with ICI alone. These findings strongly indicate that ICI injection effectively rescues primordial follicles from damage induced by SD in mice.

### 2.7 ADRB2 Mediates the Over‐Expression of KITL via the cAMP/PKA Pathway in the Activation of Primordial Follicles Induced by SD

**Figure 7:**
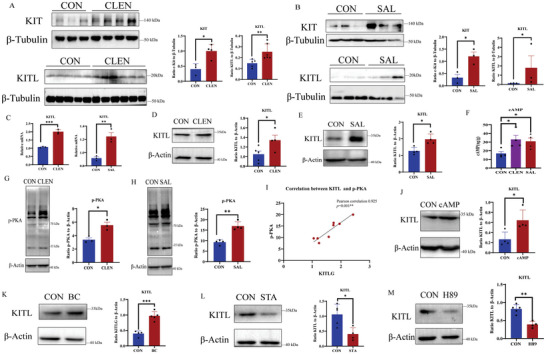
Activated ADRB2 upregulates KITL via the cAMP/PKA pathway. A) After treating mice with the ADRB2 agonist CLEN, Western blot analysis revealed high expression of KIT/KITL in ovarian tissue (KIT: CON *n* = 3, CLEN *n* = 4), (KITL: CON *n* = 5, CLEN *n* = 6). Statistical methods: KIT: Independent Samples Test; KITL: Mann‐Whitney Test. B) Similarly, after treatment with another ADRB2 agonist SAL, Western blot analysis showed high expression of KIT/ KITL in ovarian tissue (CON *n* = 3, SAL *n* = 3). Statistical methods: KIT: Independent Samples Test; KITL: Mann‐Whitney Test. C) Stimulation of the human granulosa cell line KGN with CLEN and SAL at a concentration of 1 and 100 µm, respectively, resulted in a significant upregulation of KITL mRNA expression (CON *n* = 3, CLEN *n* = 3) (CON *n* = 3, SAL *n* = 3). Statistical method: Independent Samples Test. D,E) Following treatment with CLEN and SAL, Western blot analysis revealed a significant increase in KITL protein levels in KGN cells (CON *n* = 5, CLEN *n* = 4) (CON *n* = 4, SAL *n* = 4). Statistical method: Independent Samples Test. F) After treatment with CLEN and SAL, KGN cells were collected and subjected to cAMP ELISA testing, which showed a significant increase in cellular cAMP content compared to control cells (CON *n* = 4, CLEN *n* = 4; SAL *n* = 4). Statistical method: One‐way ANOVA with LSD Test. G,H) Following treatment with CLEN and SAL, Western blot analysis showed a significant upregulation of p‐PKA levels in KGN cells (CON *n* = 3, CLEN *n* = 3) (CON *n* = 4, SAL *n* = 4). Statistical method: Independent Samples Test. I) Correlation analysis of the protein levels of KITL and p‐PKA in KGN cells revealed a strong correlation between the expression levels of these two proteins, with a Pearson correlation of 0.925 (*n* = 8, *p = *0.001**). J) After treating KGN cells with 20 µm of cAMP, the expression level of KITL significantly increased (CON *n* = 4, cAMP *n* = 4). Statistical method: Mann‐Whitney Test. K) Treatment of KGN cells with 10 nm concentration of the PKA activator BC resulted in a significant upregulation of KITL levels (CON *n* = 4, BC *n* = 5). Statistical method: Independent Samples Test. L,M) Treatment of KGN with the PKA antagonists STA and H89 in a 100 nm concentration led to a significant downregulation of KITL levels (CON *n* = 5, STA *n* = 4) (CON *n* = 5, H89 *n* = 4). Statistical method: Independent Samples Test. Data are presented as mean with SEM. In all panels, *p < 0.05, **p < 0.01, and ***p < 0.001. CON, control; CLEN, Clenbuterol; SAL, Salbutamol; BC, Bucladesine sodium; STA, Staurosporine; H89, H‐89 dihydrochloride; KITL, KIT ligand.

KITL‐KIT plays a crucial role in the initiation and activation of primordial follicles^[^ ^13^ ^]^ After treating mouse ovaries with ADRB2 agonists of either CLEN or SAL, we observed a significant increase in the protein expression levels of KIT and KITL in the mouse ovarian tissue (**Figure** **7**A,B). Immunofluorescence analysis indicated that the ADRB2 agonists, CLEN and SAL, increased KIT expression in the oocytes of ovarian primordial follicles (Figure S11A,B, Supporting Information). The ovarian granulosa cell line KGN was further treated with the ADRB2 agonists CLEN and SAL in vitro, and the results showed that both the mRNA and protein expression levels of KITL were considerably increased (Figure 7C–E).

ADRB2 locates on the cell membrane. Upon activation of the ADRB2, it stimulates the Gs subunit of the G protein family, leading to the activation of adenylate cyclase. Subsequently, ATP is converted into cAMP. A transient increase in cAMP activates PKA, which regulates various cellular processes, including differentiation, morphology, movement, secretion, neurotransmission, and gene transcription^[^ ^29^ ^]^ To investigate how the activation of ADRB2 regulates KITL in vitro, we examined the cAMP concentration and p‐PKA levels in KGN cells after treatment with ADRB2 agonists. Intriguingly, the cAMP concentration in KGN cells increased following CLEN and SAL treatment (Figure 7F), accompanied by a significant increase in p‐PKA levels (Figure 7G,H). After treatment with ADRB2 agonists, a similar expression trend was observed in ovarian tissues of mice (Figure S10B,C, Supporting Information). Correlation analysis of the protein expression levels of KITL and p‐PKA after stimulation of KGN with SAL revealed a strong positive correlation with a Pearson Correlation of 0.925 (*p = *0.001) (Figure 7I), suggesting that the activation of PKA may be a key factor in the upregulation of KITL expression. Subsequently, treatment of KGN cells with cAMP significantly increased the expression of the KITL protein (Figure 7J). Upon treatment of KGN with the PKA activator BC (bucladesine sodium), KITL levels were significantly upregulated (Figure 7K). Conversely, stimulation of KGN with the PKA antagonists staurosporine (STA) and H‐89 dihydrochloride (H89) led to the downregulation of KITL levels (Figure 7L,M). Additionally, increasing cAMP levels stimulated mTOR phosphorylation and subsequently increased KITL expression in granulosa cells (Figure S12, Supporting Information).

Collectively, these results indicated that the ADRB2 axis regulates KITL expression by activating the cAMP/PKA pathway in SD‐induced primordial follicles.

## 3 Discussion

**Figure 8:**
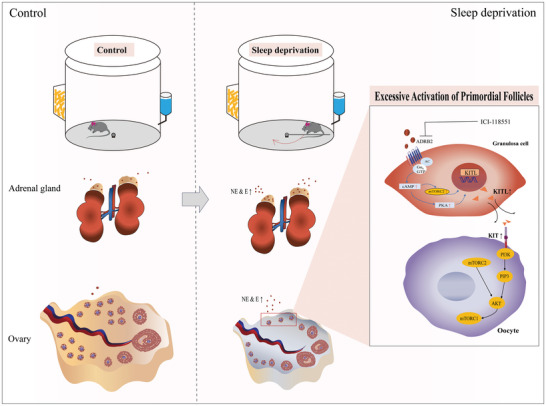
A schematic model depicting the mechanism of activation of ovarian primordial follicles induced by SD. SD leads to adrenal hyperactivity, resulting in the secretion of NE and E, which act on the ADRB2 of granulosa cells in primordial follicles of the ovary. This activation of ADRB2 triggers the cAMP/PKA and cAMP/mTOR signaling pathway in granulosa cells, leading to increased expression of KITL which initiating the activating of primordial follicles. Overactivation of primordial follicles accelerates the depletion of ovarian reserves and ultimately reduces the reproductive longevity. SD, sleep deprivation; NE, norepinephrine; E, epinephrine; ADRB2, β2‐adrenergic receptor.

Previous studies have shown that sleep is associated with female reproductive health. However, the mechanism by which SD affects ovarian function remains unknown. In this study, we found that SD leads to the loss of ovarian primordial follicles, which maintain the reserves of normal ovarian function. Intriguingly, we first found that the adrenal gland plays a vital role in the SD‐mediated stress response, which produces excessive amounts of the stress hormones NE and E and increased levels of NE/E triggers the activation of the KITL‐KIT/PI3K and mTOR pathways involved in follicle activation, leading to the loss of primordial follicles. Our data suggests that primordial follicle activation is dependent on ADRB2. ADRB2 activates the downstream kinases cAMP/PKA, thereby upregulating KITL in granulosa cells. Our study presents an intriguing mechanism through which SD affects ovarian function (**Figure** **8**).

Our study revealed for the first time that SD leads to a reduction in the number of primordial follicles in mice. Women are born with a finite pool of primordial follicles, and the number of primordial follicles in the ovarian reserve is an important determinant of the ovarian lifespan. While the majority of primordial follicles maintain a dormant state, only a small portion of primordial follicles are progressively recruited into the growing follicle pool during each cycle, which is called primordial follicle activation. Excessive activation of primordial follicles accelerates follicular pool consumption and leads to premature ovarian failure, which has important consequences for the reproductive and general health of women, including infertility.^[^ ^19^, ^30^ ^]^ Currently, the main reasons for the reduction in primordial follicles are believed to be apoptosis, iron death, and excessive activation of primordial follicles. In the present study, we did not observe either ferroptosis or apoptosis of primordial follicles in the ovaries of SD mice. However, evidence including the increased count of ovarian antral follicles, upregulated levels of serum AMH, increased protein expression levels of AMH and ZP3 in ovarian tissue, the phosphorylation levels of the PI3K/AKT and mTOR pathways associated, and Single‐cell RNA‐seq profiling associated with primordial follicle activation significantly increased, indicating that SD leads to the excessive activation of primordial follicles.

We demonstrated that SD led to the upregulation of KIT/KITL expression in mouse ovarian tissue, triggering the activation of downstream PI3K and mTOR pathways and inducing the activation of primordial follicles. The primordial follicle consists of quiescent oocytes surrounded by flattened pfGCs. GCs have been proposed as fundamental for the quality of oocytes due to their close biodynamic interrelationship. Thus the changes of mechanism occurring in GCs will necessarily affect the development of follicles. Activation of primordial follicles is a complex and coordinated process involving oocytes and pfGCs. Many molecules, including KITL,^[^ ^31^ ^]^ growth differentiation factor 9 (GDF‐9),^[^ ^32^ ^]^ and epidermal growth factor (EGF),^[^ ^33^ ^]^ are involved in the activation process of primordial follicles. KITL is the primary upstream mediator of primordial follicle activation. PI3K and mTOR signaling are the dominant pathways in pfGCs of oocytes. KITL‐KIT can activate PI3“‐kinase by directly binding to the tyr‐721 in KIT or indirectly by binding to the tyrosine‐phosphorylated adaptor protein GAB2.^[^ ^34^ ^]^ KITL is located upstream of the PI3K signaling pathway. The serine/threonine kinase AKT is downstream of PI3” kinase and is involved in KITL survival signaling.^[^ ^14^ ^]^ mTORC1 acts downstream of AKT, and mTORC1 phosphorylation promotes transcription factor expression and translation, thereby affecting cell proliferation and development.^[^ ^14^ ^]^ mTORC2/AKT pathway in oocytes regulates folliculogenesis and its inactivation causes ovarian damage.^[^ ^35^ ^]^ In Kitl‐deficient *Kitl^Sl‐t^/Kitl^Sl‐^* ^t^ mutant mice, ovarian primordial follicles failed to activate and develop further. When gene delivery technology was used to upregulate Kitl expression in the ovaries of *Kitl^Sl‐t^/Kitl^Sl‐t^* mutant mice, primordial follicles were activated and underwent further development.^[^ ^36^ ^]^ Our results showed that the cAMP/PKA pathway regulates the expression of KITL in granulosa cells. Therefore, the activation of the mTOR pathway in granulosa cells leads to increased KITL expression, which in turn activates primordial follicles.

Short‐term SD, long‐term sleep restriction, and untreated sleep disorders have profound and detrimental effects on physical and mental health, mood, and public safety. The mechanisms mediating the associations between sleep and health involves immune,^[^ ^23^, ^37^ ^]^ nerve,^[^ ^38^ ^]^ metabolic,^[^ ^39^ ^]^ and other pathways, and their dysfunction plays a determinant role in the development and progression of chronic diseases. In addition, evidence also showed that SD affects the hypothalamic‐pituitary‐adrenal (HPA) ‐axis response to psychosocial stress or produces excessive stress hormones in the serum.^[^ ^27^, ^40^ ^]^ In the present study, *Rag1* KO mice underwent SD. The number of primordial follicles and total follicles in *Rag1* KO mice still showed a considerable decrease, indicating that the loss of ovarian primordial follicles is not primarily driven by immune attack. We found that the adrenal gland plays a key role in the loss of ovarian primordial follicles caused by SD by using adrenalectomized (ADX) mice. The adrenal glands predominantly secrete four stress hormones: CORT and COR from the cortex and NE and E from the medulla, responding to the body's “fight or flight” reactions. Our investigation revealed the involvement of catecholamine hormones, specifically NE and E, produced by the adrenal glands, in this intricate process. In addition, the primary mechanisms induced by SD, particularly those involving the adrenergic receptor, are prominently observed in granulosa cells. These cells exhibit significant changes in gene expression and signaling pathways in response to SD, which aligns with their role in follicular maturation and hormone production. Disruptions of granulosa cell pathway will directly effect on the function of oocytes. Therefore, while granulosa cells may be the initial responders to SD, the repercussions are seen throughout the follicle.

NE/E binds to the adrenergic receptors expressed on the surfaces of various cells. Our investigation revealed the involvement of catecholamine hormones, specifically NE and E, produced by the adrenal glands, in this intricate process. Several studies have reported that stress increases catecholamine levels, promoting cancer cell proliferation and invasive growth. Catecholamine levels sharply increase during chronic, acute, and sustained stress responses.^[^ ^41^ ^]^ Acting on ADRB2, the catecholamine hormone activates LDHA to generate lactate, reshaping metabolic pathways in tumor cells to enhance glycolysis, thereby promoting cancer development.^[^ ^42^ ^]^ Our study found significant increases in NE and E levels after SD, which triggered a cascade response by acting on ADRB2. NE is also a neurotransmitter that originates from the central and peripheral nervous systems. Under stress conditions, activation of the sympathetic nervous system leads to neurotransmitter release, acting on ADRB2 receptors and causing rapid proliferation, differentiation, migration, and permanent depletion of quiescent melanocyte stem cells, leading to premature graying.^[^ ^43^ ^]^ The ovary is innervated by catecholaminergic nerve fibers and catecholamines are physiologically involved in the control of ovarian function.^[^ ^44^ ^]^ Although we did not observe improvement in the reduction of primordial follicles after SD following chemical ablation of sympathetic nerves, we cannot completely rule out the role of NE released by the sympathetic nervous system in this process, as NE released by the sympathetic nervous system also acts on ADRB2 receptors. CORT, the adrenal cortex hormone in the HPA axis,^[^ ^45^ ^]^ also plays an important role in stress; however, in our study, no statistically significant differences were found in CORT and COR in mouse serum after SD treatment, so we did not further explore the role of the HPA axis.

The mechanism by which the activation of ADRB2 regulates KITL expression in the ovaries is unclear. Notably, the ovarian tissue is abundant in adrenergic receptors, which exhibit swift and precise responsiveness to fluctuations in catecholamine hormones. ADRB2 is present in GCs of primordial, primary, secondary, and preantral follicles, not only in mice but also in human eggs or monkeys, and GCs staining of primary follicles is strong^[^ ^46^ ^]^ We found that treatment of KGN cells with ADRB2 agonists activates the downstream cAMP/PKA pathway. Using PKA activators and inhibitors, the expression of KITL was upregulated and downregulated, respectively, further confirming that PKA activation is an upstream pathway that regulates KITL. Transcriptional changes induced by PKA often promote cell differentiation at the expense of cell proliferation and coordinate transcriptional responses to stress or homeostatic disturbances.^[^ ^47^ ^]^ Intriguingly, this is the first study to demonstrate that ADRB2/cAMP/PKA controls the generation of KITL during the activation of primordial follicles during SD.

To observe the effects of SD on ovaries more significantly, we employed a complete SD model. However, this model does not account for the reduction in daily sleep time that often occurs in real life. The findings of this study provide directions and insights for the next step in chronic partial SD model research. Although we demonstrated that ADRB2 signaling regulates KITL through the downstream cAMP/PKA inhibitor pathway, the essential role of this pathway in SD is yet to be validated in vivo. In this study, we also observed a reduced corpus luteum count (Figure S13A,B, Supporting Information), estrous cycle disruption (Figure S13C,D), cellular apoptosis (Figure S14), and ferroptosis (Figure S5C, Supporting Information) in mouse ovaries after SD; however, further investigation was not pursued. This will be further explored in future studies. In future clinical studies, we intend to employ more innovative, convenient, and effective methods, such as the metabolic fingerprinting of follicular fluid (MFFF) detected by particle‐assisted laser desorption/ionization mass spectrometry (PALDI‐MS)^[^ ^48^ ^]^ to further evaluate the impact of sleep loss or deprivation on female reproductive capacity in humans.

In conclusion, our research sheds light on the mechanism by which SD leads to adrenal gland hyperactivity, resulting in an increased secretion of catecholamine hormones. This, in turn, leads to the overactivation of ADRB2 receptors, subsequently triggering the overexpression of KIT/KITL via the cAMP/PKA pathway and the consequential overactivation of primordial follicles, ultimately resulting in the loss of the ovarian reserve.

## 4 Experimental Section

### Mice

The 6–8 to week‐old C57BL/6J strain of female mice originated from the Lingchang Organism, while 6 to 8 week‐old *Rag1* KO female mice were obtained from the Shanghai Model Organisms Center, Inc. All mice were reared under a standardized light/dark cycle from 8 a.m. to 6 p.m., with unrestricted access to water and food.

SD was administered using an innovative system (SA109; Cyons, China) designed for this purpose. The system utilizes a cage‐based approach and employs an adjustable frequency deprivation rod that scans back and forth along the bottom of the cage, creating a barrier‐free environment for SD experiments. This system effectively simulates a natural living environment for animals and ensures access to sufficient food and water. Prior to commencement of the experiment, C57BL/6J female mice aged 6–8 weeks were randomly assigned to three groups. The mice were introduced into the SD apparatus for an initial period of 2 days. Following this adaptation phase, the experimental groups were subjected to SD for 0, 3, and 6 consecutive days with the rotational speed of the deprivation rod set at 6 RPM. The mice were exposed to a standardized photoperiod with constant temperature (25 °C), and stable humidity (≈50% relative humidity), consisting of 10 h of light (beginning at 8:00 A.M.) and 14 h of darkness.

### Mouse Drug Treatments

Freshly prepared solutions of NE and E were prepared by dissolving them in a mixture of 0.1% ascorbic acid and 0.9% normal saline (NS). For the NE treatment groups, mice received daily intraperitoneal injections of 0.2 and 2 mg kg^−1^ for 6 days, respectively. Similarly, mice in the E treatment groups received daily intraperitoneal injections of 0.2 and 2 mg kg^−1^ of the drug once a day for 6 days.^[^ ^49^ ^]^ Control mice were injected with an equivalent volume of the ascorbic acid and NS solvent mixture to ensure consistency in treatment conditions. ADRB2 receptor agonist, CLEN (Macklin, C805465), was dissolved in the drinking water of mice and administered at a dose of 5 mg kg^−1^ for 6 days, while the control group received regular drinking water. Another ADRB2 agonist SAL (MCE, AH‐3365), dissolved in DMSO, was administered via intraperitoneal injections (0.2 mg kg^−1^) daily for 6 days, while the control group received an equivalent volume of DMSO solution. The ADRB2 antagonist ICI (MCE, HY‐13951), dissolved in DMSO, was administered via daily intraperitoneal injections (5 mg kg^−1^) for 6 days.^[^ ^50^ ^]^ The control group and the SD6D group were concurrently injected with equivalent volumes of DMSO.

### ADX

Female C57BL/6J mice aged 6–8 weeks were randomly divided into two groups: one group underwent a sham operation (sham group), while the other group underwent bilateral adrenal resection (ADX group). Following anesthesia, the mice had the fur on their back shaved, and the skin was disinfected with 75% alcohol. A bilateral longitudinal incision was made 1 centimeter (cm) from the left and right sides of the midline, just below the ribcage's lower margin. The muscles were carefully separated to access the abdominal cavity, and the internal organs were gently moved aside using a saline‐soaked cotton ball held with small forceps. The pale yellow adrenal glands, distinct from the surrounding fatty tissue, were located just above the kidney. With surgical forceps, the adrenal gland was delicately removed. The same procedure was repeated for the removal of the right adrenal gland. Afterward, the muscle and skin were sutured. The removed adrenal glands were preserved for reference. The sham group underwent a procedure similar to the experiment but without adrenal gland removal. Following the removal of the adrenal glands, the mice were recovered for 1–2 weeks before further experimentation.

### Ethics Statement

All the animal experiments were performed in compliance with the General Code of Animal Welfare of the Ministry of Science and Technology. The ethical aspects of all experimental procedures were approved by the Animal Protection and Use Committee of Shanghai Jiao Tong University School of Medicine (Protocol B‐2019‐013). The license number for the use of experimental animals was SYXK‐2018‐0027, approved by the Shanghai Science and Technology Commission.

### Follicle Count

The collected ovaries were fixed in 4% paraformaldehyde and subsequently embedded in paraffin. After sectioning the tissue into continuous 5 µm‐thick slices, every fifth section was chosen for sequential HE staining. HE staining was employed to differentiate and quantify follicles at different stages by two independent researchers. Each stage of follicle was counted according to established definitions.^[^ ^51^ ^]^ Primordial follicles, classified as Grade I oocytes, are characterized by a single layer of flat squamous granular cells. As these cells transform into a single layer of cuboidal granular cells, they become primary follicles. Secondary follicles are identified by having two or more layers of granular cells, with no follicular fluid between these cells. With further development, the granular cell layer increases, leading to the production of follicular fluid and the formation of a follicular cavity, resulting in antral follicles. Primordial follicles, primary follicles, secondary follicles, and antral follicles are collectively referred to as total follicles; primary follicles, secondary follicles, and antral follicles are referred to as developing follicles.

### Single Cell Isolation

For each sample, ovarian tissues from two mice of the same group were pooled and processed together. The ovaries were initially preserved in Miltenyi Tissue Storage Solution at low temperature and then transported to the laboratory for preparation of cell suspensions. Prior to dissociation, the tissues were washed with phosphate‐buffered saline (PBS) containing 0.04% bovine serum albumin (BSA). The ovaries were then finely minced and incubated in a mixture of enzymes (collagenase IV, dispase, and DNase) at 37 °C in a shaking water bath for 30 min. The digestion process was halted by adding DMEM containing BSA. The resulting suspension was passed through a 70 µm cell strainer (Miltenyi). Red blood cells were lysed using Red Blood Cell Lysis Solution (Miltenyi), and the cells were subsequently resuspended in PBS with 0.04% BSA. The suspension was then filtered through a 40 µm cell strainer (Miltenyi). The concentration and viability of the cells were assessed using the AcridineOrange/Propidiumiodide method.

### Single‐cell RNA Seq and Analysis

For single‐cell RNA sequencing, cell suspensions were processed using Chromium microfluidic chips from 10x Genomics. Depending on the project requirements, either the 3′ v2 or v3 chemistry was utilized for loading cells into the Chromium Controller, which assigns unique barcodes to individual cells. Following barcoding, RNA from the cells was reverse‐transcribed, and sequencing libraries were constructed using the Chromium Single Cell 3′ reagent Kits (v2 or v3) as the manufacturer's protocol (10x Genomics). Sequencing was performed with Illumina (HiSeq 2000 or NovaSeq, depends on project) according to the manufacturer's instructions (Illumina). Initial quality control of the raw sequencing reads was performed using fastp, providing basic statistical assessments. Raw reads were demultiplexed and mapped to the reference genome by 10 X Genomics Cell Ranger pipeline (https: //support.10xgenomics.com single‐cell‐geneexpression/software/pipelines/latest/what‐is‐cell‐ranger) using default parameters. Subsequent single‐cell analyses were primarily performed using Cell Ranger and Seurat tools, unless otherwise specified. The digital gene expression matrices were created by counting unique molecular identifiers for each gene and cell barcode filtered by Cell Ranger. For further filtering with Seurat, a gene was considered expressed if detected in more than three cells, and each cell was required to express at least 200 genes. Additionally, any contaminant cells were filtered out. The cell ranger reanalyze function was utilized for downstream analysis, including dimensionality reduction, clustering, and gene expression profiling, by reprocessing the feature‐barcode matrices generated from cell ranger count or cell ranger aggr, using the default parameters provided by Cell Ranger. More details were provided in the Supporting Information. Single‐cell RNA sequencing was conducted by Novogene.

### mRNA‐Sequence

Ovarian tissue mRNA sequencing was conducted by Novogene. High‐quality RNA samples from ovarian tissue were subjected to sequencing on an Illumina NovaSeq 6000 platform. A reference genome index was constructed using HISAT2 (v2.0.5), and the paired‐end clean reads were mapped to the reference genome using HISAT2 (v2.0.5). Differential expression analysis between two comparison groups was conducted using DESeq2 software (1.20.0). Gene Ontology (GO) and Kyoto Encyclopedia of Genes and Genomes (KEGG) enrichment analyses of differentially expressed genes were performed using clusterProfiler (3.8.1) software. More details were provided in the Supporting Information.

### Detection of Serum Sex Hormones

Serum AMH levels were measured using the Mouse anti‐Mullerian hormone (AMH) ELISA kit from Cusabio (Cusabio, CSB‐E13156m). Levels of E2 and FSH were assessed using the 17 beta Estradiol ELISA Kit (Abcam, ab108667) and the FSH ELISA kit (Xinle, XL‐EM0427), respectively. All procedures were performed according to the manufacturers' instructions provided in the kit manuals.

### Detection of Serum Stress Hormones

Serum was collected from each mouse following the specified treatments, and serum was obtained through static centrifugation for further analysis. For the detection of NE and CORT, enzyme‐linked immunosorbent assay (ELISA) kits from Cusabio (Cusabio, CSB‐E07870m) and Sangon (Sangon, D721183‐0096) were used respectively. Each well received 50 µL of serum, and the assays were conducted following the manufacturers' instructions. E was detected using a Finetest ELISA Kit (Finetest, EU2563), with 10 µL of serum added to per well. Cortisol (COR) concentrations were determined using the Mouse Cortisol ELISA Kit (FineTest, EM1721), adhering strictly to the protocol provided by manufacturer.

### Western Blotting

Ovarian tissue samples were lysed using RIPA buffer (Beyotime, P0013B) and supplemented with Protease and phosphatase inhibitor cocktail (Beyotime, P1045). For cells, 100–200 µL RIPA with Protease and phosphatase inhibitor cocktail was added to a six‐well plate containing cultured cells and cells were thoroughly lysed on a shaker. Protein concentration in each sample was determined using Beyotime's protein concentration Kit (Beyotime, P0012). Samples were then heated and mixed with SDS‐PAGE protein sample loading buffer (5X) (Beyotime, P0286). Proteins were separated by SDS‐PAGE, transferred onto a polyvinylidene fluoride membrane (PVDF), and blocked with 5% skim milk (Epizyme, PS112) in 1X TBS with Triton X‐100 (Epizyme, PS103). Primary antibodies were applied at a dilution of 1:1000 using Dilution Buffer (Beyotime, P0023A). HRP‐conjugated β‐actin (Yeasen, 30103ES60), and HRP‐conjugated β‐tubulin (Yeasen, 30303ES50) served as Loading Controls, diluted to a concentration of 1:10000 before use. Goat Anti‐Rabbit IgG H&L (HRP) (Abcam, ab6721) and Goat Anti‐Mouse IgG H&L (HRP) (Abcam, ab6789) were applied at a concentration of 1:10000. Image development was carried out using Immobilon Western Chemiluminescent HRP Substrate (Millipore, WBKLS0500), and protein content analysis was performed using ImageJ software. The primary antibodies used in the experiment were: ADRB2 (Abcam, ab182136), AMH (Abcam, ab103233), ZP3 (Santa, sc‐398359), p‐PI3K p85 ^Tyr458^/p55 ^Tyr199^ (CST, 4228T), PI3K (Abcam, ab191606), p‐AKT ^Ser473^ (CST, 4060T), AKT (CST, 4691T), mTOR (Abcam, ab32028), p‐mTOR ^Ser2481^ (Affinity, AF3309), p‐mTOR ^Ser2448^ (Affinity, AF3308), KIT (Abcam, ab32363), KITL (Abcam, ab64677), KITL (Abcam, ab52603) and p‐PKA (CST, 9624S). Anti‐AMH antibody was diluted in a 1:250 and other primary antibodies were diluted in a 1:1000 dilution buffer (Beyotime, P0023A).

### Immunofluorescence

Ovarian tissue samples were initially fixed overnight with 4% paraformaldehyde and subsequently replaced with 30% sucrose in PBS for dehydration for two days, followed by embedding and slicing using optimal cutting temperature (OCT) compound (Sakura, 4583) at a thickness of 10 micrometers. Afterward, the sections were washed twice on a horizontal shaker for 5 min at the low speed. A blocking buffer consisting of 3% BSA, 5% normal goat serum (NGS), and 10% Triton in PBS was used for tissue permeabilization and blocking, carried out in a 37 °C chamber. Following this, the blocking buffer was removed, and a droplet of primary antibody was applied to the tissue to ensure full coverage, after which it was incubated at 4 °C overnight. The secondary antibody was applied at room temperature for a 2‐h incubation period. The specific primary antibodies diluted at 1:400 were used for this process, including TH (Abcam, ab137869), KIT (CST, 3074T), p‐AKT ^Ser473^(Affinity, AF0016), p‐mTOR ^Ser2481^ (Affinity, AF3309), p‐FOXO3a^Ser253^ (Affinity, AF3020), ADRB2 (Abcam, ab182136). Goat Anti‐Rabbit IgG H&L (Alexa Fluor 488) and Goat anti‐Rabbit IgG (H + L) Alexa Fluor 555 (ThermoFisher Scientific, A‐21428) were diluted at 1:500 for secondary antibody incubation. Then the slides were sealed with mounting medium containing DAPI (Abcam, ab104139). Finally, the tissue sections were observed under a fluorescence microscope.

### Cell Culture

The human ovarian granulosa tumor cell line KGN, generously provided by the Li Laboratory, was cultivated in a medium containing 10% fetal bovine serum (FBS) and 1% penicillin and streptomycin in DMEM/F12 medium. Cell culture was maintained at 37 °C in a 5% carbon dioxide incubator. In the experimental setup, cAMP (MCE, HY‐B1511) the ADRB2 agonists CLEN (Macklin, C805465), and SAL (MCE, AH‐3365) and were dissolved in DMSO at a concentration of 20, 1, and 100 µm, respectively. The control group received an equivalent volume of DMSO and was treated for 24 h. After treating the cells with 10 nm concentration of the PKA activator BC, 100 nm concentration of the PKA antagonist STA and another PKA antagonist H89 in a 100 nm concentration for 2 h, cells were collected for further use. KGN cells were treated with 1 µm of the mTOR agonist MHY1485 (MCE, HY‐B0795), and 100 nm of the mTOR antagonist Rapamycin (MCE, HY‐10219) for 24 h, after which the cells were collected. All cell experiments were conducted using six‐well plates (Corning, 3516), with each well seeded with 1 × 10^6^ cells. The cell density was ≈80% before drug treatment.

### Ovary Culture

Neonatal C57BL/6J female mice, 6 days old, were used. Under sterile conditions and a stereomicroscope, the ovaries were microsurgically dissected from the mice using cold PBS. The isolated ovaries were placed in a cell culture insert within a 6‐well culture dish (Corning, 3422) and cultured for 4 days. The culture medium used was DMEM/F12 supplemented with 10% FBS, Insulin‐Transferrin‐Selenium‐Sodium Pyruvate (ITS‐A) (100X) (Gibco, 51 300 044), and 1% penicillin and streptomycin. The culture was maintained at 37 °C with 5% CO_2_ and saturated humidity. For the ovarian culture, the treatment group was administered KITL at a concentration of 500 ng mL^−1^ (PeproTech, 250‐03), while the control group received an equivalent volume of the solvent. The culture medium was changed every two days.

### RNA Extraction

In a six‐well plate, cells were washed twice with PBS, followed by the addition of 1 mL Trizol. Gently scraping the cells, the Trizol mixture was then aspirated using a pipette and transferred to a 1.5 mL centrifuge tube. After standing at room temperature for 5 min, 0.2 mL chloroform was added, and the tube was vigorously shaken for ≈15 s. Centrifugation at 2–8 °C, 12 000 g for 10 min resulted in three layers: a red phenol‐chloroform phase at the bottom, a clear aqueous phase on top, and a white DNA precipitate in the middle. The upper aqueous phase was transferred to a new EP tube, and 0.5 mL isopropanol was added, followed by a 10‐min incubation and subsequent centrifugation. Washing with 75% ethanol was performed twice, followed by centrifugation and air‐drying of the RNA pellet. Subsequently, the RNA was reconstituted after dissolution.

### qPCR

The extracted RNA was subjected to a DNA removal reaction. The reverse transcription of RNA was carried out following the instructions of the PrimeScript RT reagent Kit with gDNA Eraser (TAKARA, RR047A). The qPCR detection system was prepared using the QuantiNova SYBR Green PCR Kit (Qiagen, 208 054), and real‐time PCR was conducted on an ABI 7500 real‐time PCR instrument (Applied Biosystems, QuantStudio 7 Flex). The sequences of the PCR primers are provided in the experimental section of the Supporting Information. The Forward primer was used to amplify the forward segment of the target gene, while the Reverse primer was used to amplify the reverse segment of the target gene.

### cAMP Assay

After treatment, KGN cells were washed three times in PBS and lysed on ice using RIPA lysis buffer (Beyotime, P0013B). Subsequently, the cells were scraped off. Cold‐thaw cycles were performed twice using liquid nitrogen and a 37 °C water bath, followed by centrifugation at 600 × g for 10 min to collect the supernatant. The protein concentration of each sample was determined using NanoDrop 2000/2000c Spectrophotometers (Thermo Scientific) and recorded. cAMP measurement was conducted using a cAMP detection assay Kit (Elabscience, E‐EL‐0056), following the manufactuer's instructions. The cAMP concentration calculated from the ELISA assay Kit was converted to the amount of cAMP per gram of protein in the sample (ng/g) based on the determined protein concentration.

### Data Analysis

All data were analyzed using IBM SPSS (Version 26) for Windows (Chicago, IL, USA). Both visual inspection and formal normality tests were employed to verify the normal distribution of the data. When comparing two sets of independent samples, the choice between a t‐test or a non‐parametric test depended on whether the data exhibited a normal distribution and homogeneity of variances. For comparisons involving multiple groups of independent samples, one‐way analysis of variance was used to assess the differences among the groups. In cases in which the data did not conform to a normal distribution, nonparametric tests were used. Two‐tailed *p* values were calculated and statistical significance was defined as *p* < 0.05. GraphPad Prism was used for data visualization and graphical representations.

## Conflict of Interest

The authors declare no conflict of interest.

## Author Contributions

D.L. and L.W. conceived and designed the project. L.W. completed most of the experiments, wrote and revised the manuscript. H.H. and Q.Z. participated in part of the animal and cell experiments. C.X. participated in the ovarian follicle counting experiment. Q.Z., Q.W., and J.H. provided intellectual input. D.L. provided intellectual input and revised the manuscript. All authors have read and approved the final version.

## Supporting information

Supporting Information

## Data Availability

The data that support the findings of this study are available from the corresponding author upon reasonable request.
