# Supplementary material for: Sleep Deprivation Triggers the Excessive Activation of Ovarian Primordial Follicles via β2 Adrenergic Receptor Signaling
Source: Adv Sci (Weinh). 2024 Sep 4;11(41):2402393. doi: 10.1002/advs.202402393 (PMC11538700; doi:10.1002/advs.202402393)

ADVANCED SCIENCE
Open Access

Supporting Information

for *Adv. Sci.*, DOI 10.1002/advs.202402393

Sleep Deprivation Triggers the Excessive Activation of Ovarian Primordial Follicles via β2 Adrenergic Receptor Signaling

*Lichun Weng, Hanqing Hong, Qinyu Zhang, Chengqi Xiao, Qiuwan Zhang, Qian Wang, Ju Huang and Dongmei Lai**

Experimental Section

Using Water Platforms for Sleep Deprivation Model

The experiment used 6-8 week old C57BL/6J female mice. Water platforms were used to create a sleep deprivation model in mice. Mice were placed on these platforms, where they could move between columns and had access to water and ample food. When muscle tone was lost during sleep, they would touch the water and wake up. The mice were introduced to the water platform cages 2 days in advance for acclimatization. After the acclimatization phase, control group mice had their cages lined with normal bedding, while the sleep deprivation group had their cages filled with an equivalent volume of water. After 1 day, the mice were weighed, and their ovarian tissues and serum were collected for further experiments.

Generation and Analysis of Single-Cell Transcriptomes

Cell ranger count takes FASTQ files performs alignment, filtering, barcode counting, and UMI counting. It uses the Chromium cellular barcodes to generate feature-barcode matrices, determine clusters, and perform gene expression analysis. The count pipeline can take input from multiple sequencing runs on the same GEM well. Add -nosecondary option to skip secondary analysis of the feature-barcode matrix (dimensionality reduction, clustering and visualization) When doing large studies involving multiple GEM wells, run cell ranger count on FASTQ data from each of the GEM wells individually, and then pool the results using cell ranger aggr: cell ranger aggr aggregates outputs from multiple runs of cell ranger count, normalizing those runs to the same sequencing depth and then recomputing the feature-barcode matrices and analysis on the combined data. The aggr pipeline can be used to combine data from multiple samples into an experiment-wide feature-barcode matrix and analysis.
Before proceeding with secondary analysis of gene expression, we conducted a second round of quality control using Seurat. Subsequent analysis of cell ranger reanalyze and Seurat were all performed basing on this output gene expression matrix.

Secondary Analysis of Single-Cell Gene Expression and Enrichment

Cell ranger reanalyze takes feature-barcode matrices produced by cellranger count or cell ranger aggr and reruns the dimensionality reduction, clustering, and gene expression algorithms using cellranger default parameter settings.

Seurat**:** The Seurat package was used to normalise data, dimensionality reduction, clustering, differential expression. we used Seurat alignment method canonical correlation analysis (CCA) [Nat. Biotechnol. 36, 411– 420 (2018).] for integrated analysis of datasets. For clustering, highly variable genes were selected and the

principal components based on those genes used to build a graph, which was segmented with a resolution of 0.6.

Global Analysis Between Samples**:** Based on filtered gene expression matrix by Seurat, between samples differential expression analysis was carried out using the edgeR package to obtain zone-specific marker genes.

Enrichment analysis of marker genes**:** Gene Ontology (GO) enrichment analysis of marker genes was implemented by the clusterProfiler R package, in which gene length bias was corrected. GO terms with corrected Pvalue less than 0.05 were considered significantly enriched by marker gene.

KEGG is a database resource for understanding high-level functions and utilities of the biological system, such as the cell, the organism and the ecosystem, from molecular-level information, especially large-scale molecular datasets generated by genome sequencing and other high-through put experimental technologies (http://www.genome.jp/kegg/). We used clusterProfiler R package to test the statistical enrichment of marker genes in KEGG pathways.

Reactome pathway-based analysis of marker genes was implemented by the ReactomePA R package. REACTOME is an open-source, open access, manually curated and peer-reviewed pathway database (https://reactome.org/) Protein-Protein Interaction networks (PPI) analysis of marker genes was implemented by the STRINGdb R package.

mRNA-Seq Data Analysis Process

1. Data Quality Control

The process begins with converting image data from high-throughput sequencers into sequence data (reads) through CASAVA base calling. The raw sequencing data often contains a small percentage of reads with sequencing adapters or low-quality sequences. To ensure the reliability and quality of data analysis, it is essential to filter these raw reads. The filtering steps include:

- Removing reads with adapters.

- Excluding reads containing undetermined bases (N).

- Eliminating low-quality reads where bases with Qphred ≤ 20 constitute more than 50% of the read length.

Subsequently, we calculate Q20, Q30 scores, and GC content for the clean data. All downstream analyses are performed on this high-quality clean data.

2. Alignment to the Reference Genome

The reference genome and gene model annotation files are downloaded directly from genome databases. Using HISAT2 (v2.0.5), we construct an index for the reference genome and align the paired-end clean reads to it. HISAT2 is chosen because it supports the generation of spliced alignments based on gene model annotation files, providing better alignment results compared to non-spliced aligners.

3. Quantification of Gene Expression Levels

Gene expression levels are quantified using feature Counts (v1.5.0-p3), which counts the number of reads mapped to each gene. The expression level for each gene is then calculated as FPKM (Fragments Per Kilobase of transcript per Million mapped reads), accounting for sequencing depth and gene length. FPKM is a widely used metric for estimating gene expression levels.

4. Differential Expression Analysis

For samples with biological replicates, differential expression analysis between comparison groups is performed using DESeq2 (v1.20.0). DESeq2 employs a statistical model based on the negative binomial distribution to identify differentially expressed genes from count data. P-values are adjusted using the Benjamini-Hochberg method to control the false discovery rate (FDR). Genes with adjusted p-values (padj) ≤ 0.05 and $|\log2(\text{fold change})| \geq 1$ are considered significantly differentially expressed.

For samples without biological replicates, edgeR is used. Before differential expression analysis, read counts are normalized using a scaling factor in the edgeR package. Differential expression analysis between conditions is then performed with edgeR (v3.22.5). P-values are also adjusted using the Benjamini-Hochberg method, with padj ≤ 0.05 and $|\log2(\text{fold change})| \geq 1$ set as the thresholds for significant differential expression.

5. Enrichment Analysis of Differentially Expressed Genes

Gene Ontology (GO) enrichment analysis of differentially expressed genes is conducted using clusterProfiler (v3.8.1), with adjustments for gene length bias. GO terms with an adjusted p-value < 0.05 are considered significantly enriched.

KEGG pathway analysis involves evaluating the enrichment of differentially expressed

genes in biological pathways using clusterProfiler. KEGG provides a resource for understanding high-level functions of biological systems from molecular-level information.

Reactome pathway analysis and Disease Ontology (DO) enrichment analysis are performed similarly. Reactome focuses on reactions and biological pathways, while DO connects gene functions with human diseases. Both analyses use clusterProfiler with an adjusted p-value < 0.05 as the significance threshold. DisGeNET enrichment analysis identifies human disease-related genes and follows the same threshold criteria for significance.

Sympathetic Ablation in Mice

To achieve sympathetic ablation, we utilized 6-hydroxydopamine bromide (6-OHDA) (MCE, HY-B1081A) solution to disrupt the sympathetic nervous system in mice. 6-OHDA was dissolved in a 0.4% ascorbic acid (Servicebio, GC301015) PBS solution and was prepared for use. In the drug treatment group, mice received intraperitoneal injections of 6-OHDA at doses of 2 mg/kg or 200 mg/kg the day prior to SD. The CON and SD6D groups received equivalent volumes of a 0.4% vitamin C PBS solution.

Detection of Estrous Cycle in Mice

The estrous cycles of the mice were examined daily at 8 a.m. For the procedure, a 10 μLvolume of sterile saline solution was carefully injected into the vagina of each mouse to ensure the injection process remained sterile. Following the injection, the solution was gently aspirated and placed onto a microscope slide to prepare a vaginal smear. Once the smear had dried naturally, a rapid Giemsa stain was applied. After completing the staining process, the smear was examined under a microscope to determine the estrous cycle stage based on the following morphological criteria: Proestrus: The smear displays a large number of nucleated epithelial cells, which are typically round or oval in shape. Estrus: The smear is characterized by numerous large, flat, anucleate (without nuclei) keratinized squamous cells with irregular edges. There are no leukocytes or nucleated epithelial cells visible. Metestrus: The smear shows a mixture of keratinized epithelial cells, nucleated epithelial cells, and leukocytes (white blood cells). Diestrus: The smear predominantly contains leukocytes, often mixed with mucus. There are fewer epithelial cells of any kind.

TUNEL Assay

Ovarian tissue sections were deparaffinized and hydrated using a gradient of ethanol, followed by a 5-minute wash in distilled water. The tissue was circumscribed using a

PAP Pen, and 20 μg/ml Proteinase K (Beyotime, ST532) diluted in immunostaining wash solution (Beyotime, P0106) was applied to the tissue, followed by an incubation at room temperature for 30 minutes. The tissue was then washed three times with PBS, each time for 5 minutes. TUNEL detection solution was prepared according to the Kit instructions (Beyotime, C1089), applied to the tissue, and incubated in the dark at 37°C for 60 minutes. After washing with PBS, the slides were sealed with mounting medium containing DAPI (Abcam, 104139). The specimens were observed and photographed under a fluorescence microscope. Ovarian sections from chemotherapy-induced POI mice were used as a positive control. These sections were kindly provided by Dr. Cao.

Prussian Blue Iron Staining

Ovarian tissue sections were dewaxed to water, followed by a 5-minute wash in distilled water. The Potassium Ferrocyanide Solution and Acid Solution from the Kit (Yeasen, 60533ES20) were mixed in specific volumes to create Perls stain. The sections were immersed in Perls stain, left to stain for 1 hour, and then thoroughly rinsed with distilled water for 2-5 minutes. The sections were then placed in Nuclear Fast Red staining solution, lightly stained for 1-5 minutes, and rinsed with tap water for 30 seconds. After dehydration and clearing, neutral resin was used for mounting, and observations were made under the microscope. Ovarian sections from chemotherapy-induced POI mice were used as a positive control. These sections were kindly provided by Dr. Cao.

Design of the qPCR primer sequences

The sequences of the PCR primers designed using the KITL gene as a template. The primer sequences are as follows: Forward primer’ 5'-AATCCTCTCGTCAAAACTGAAG’-3'; Reverse primer’ 5'-CCATCTCGCTTATCCAACAATG’-3'. The other primer sequences of a reference gene GAPDH are as follows: Forward primer’ 5'-GTCTCCTCTGACTTCAACAGC’-3'; Reverse primer’ 5'- ACCACCCTGTTGCTGTAGCCA’-3'.

Supplement Results

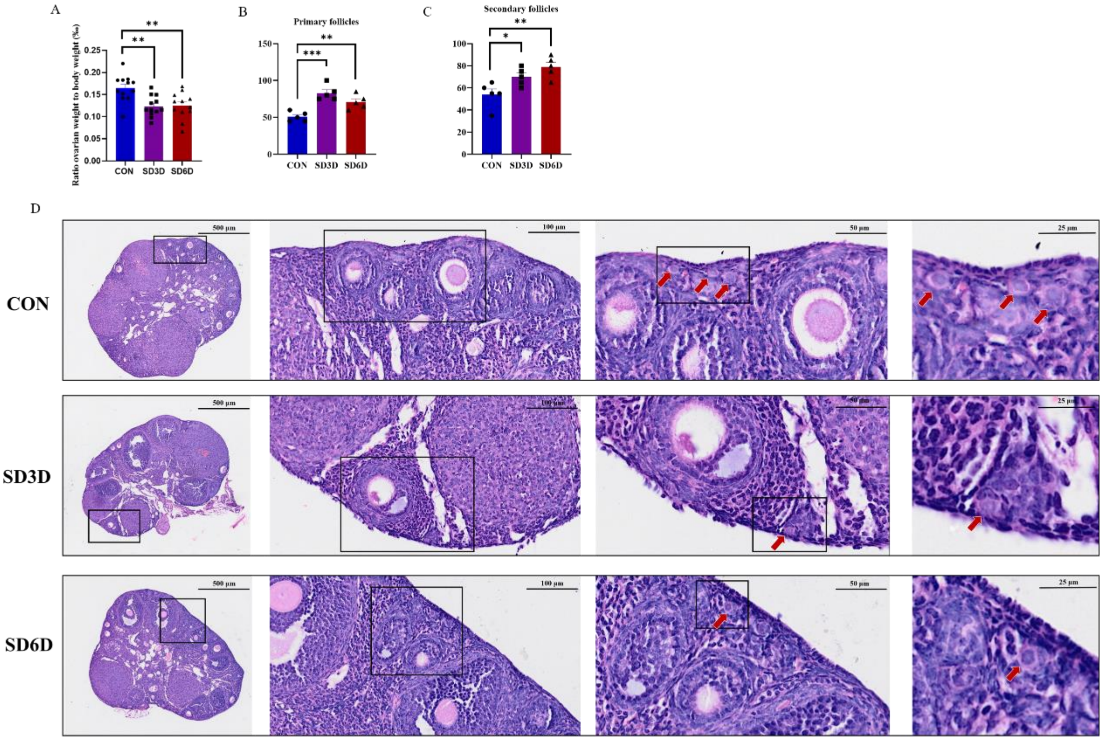

Figure S1. Hematoxylin and eosin (HE) staining image of ovarian tissue

A) Ratio of ovarian weight to body weight in mice after 0, 3, 6 days of SD (CON n = 12; SD3D n = 12; SD6D n = 12). Statistical method: One-way ANOVA with LSD Test.

B, C) The number of primary and secondary follicles in mice after SD (CON n = 12; SD3D n = 12; SD6D n = 12). Statistical method: One-way ANOVA with LSD Test.

D) Histological images of mouse ovarian tissue sections stained with HE after SD treatment for 0, 3, and 6 days, with red arrows indicating primordial follicles. Scale bar, 500, 100, 50, and 25 μm.

Data are presented as mean with SEM. In all panels, *$p < 0.05$, and ***$p < 0.001$, ns indicates not significant. CON, control; SD, sleep deprivation.

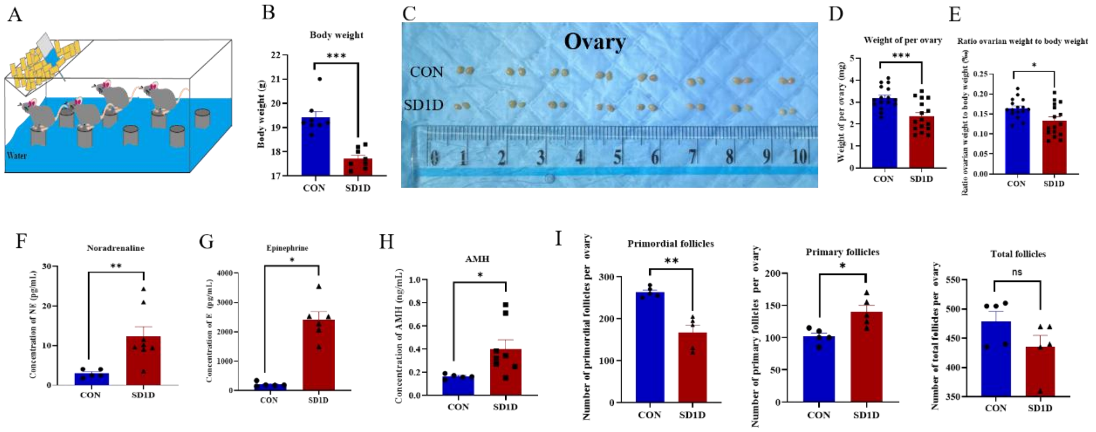

Figure S2 SD on water platforms induces the loss of primordial follicles

A) Schematic diagram of water platform apparatus. B) Significant decrease in body weight of mice after SD (CON n = 8; SD1D n = 8). Statistical method: Mann-Whitney Test. C, D) Representative images of mouse ovaries after SD treatment, showing a significant reduction in ovarian weight in sleep-deprived mice (CON n = 16; SD1D n = 16). Statistical method: Independent Samples Test. E) Ratio of ovarian weight to body weight in mice after 0, and 1 day of SD (CON n = 16; SD1D n = 16). Statistical method: Mann-Whitney Test. F) The serum NE levels in mice significantly increased after sleep deprivation (CON n = 5; SD1D n = 8). Statistical method: Mann-Whitney Test. G) The serum E levels in mice significantly increased after sleep deprivation (CON n = 5; SD1D n = 6). Statistical method: Mann-Whitney Test. H) The serum AMH levels in mice significantly increased after sleep deprivation (CON n = 5; SD1D n = 8). Statistical method: Mann-Whitney Test. I) Number of primordial follicles, primary follicles, total follicles count in mice after SD treatment (CON n = 5; SD1D n = 5). Statistical methods: Primordial follicles: Mann-Whitney Test; Primary follicles: Independent Samples Test; Total follicles: Mann-Whitney Test. Data are presented as mean with SEM. *$p < 0.05$, **$p < 0.01$, ***$p < 0.001$, and ns indicates not significant. CON, control; SD, sleep deprivation.

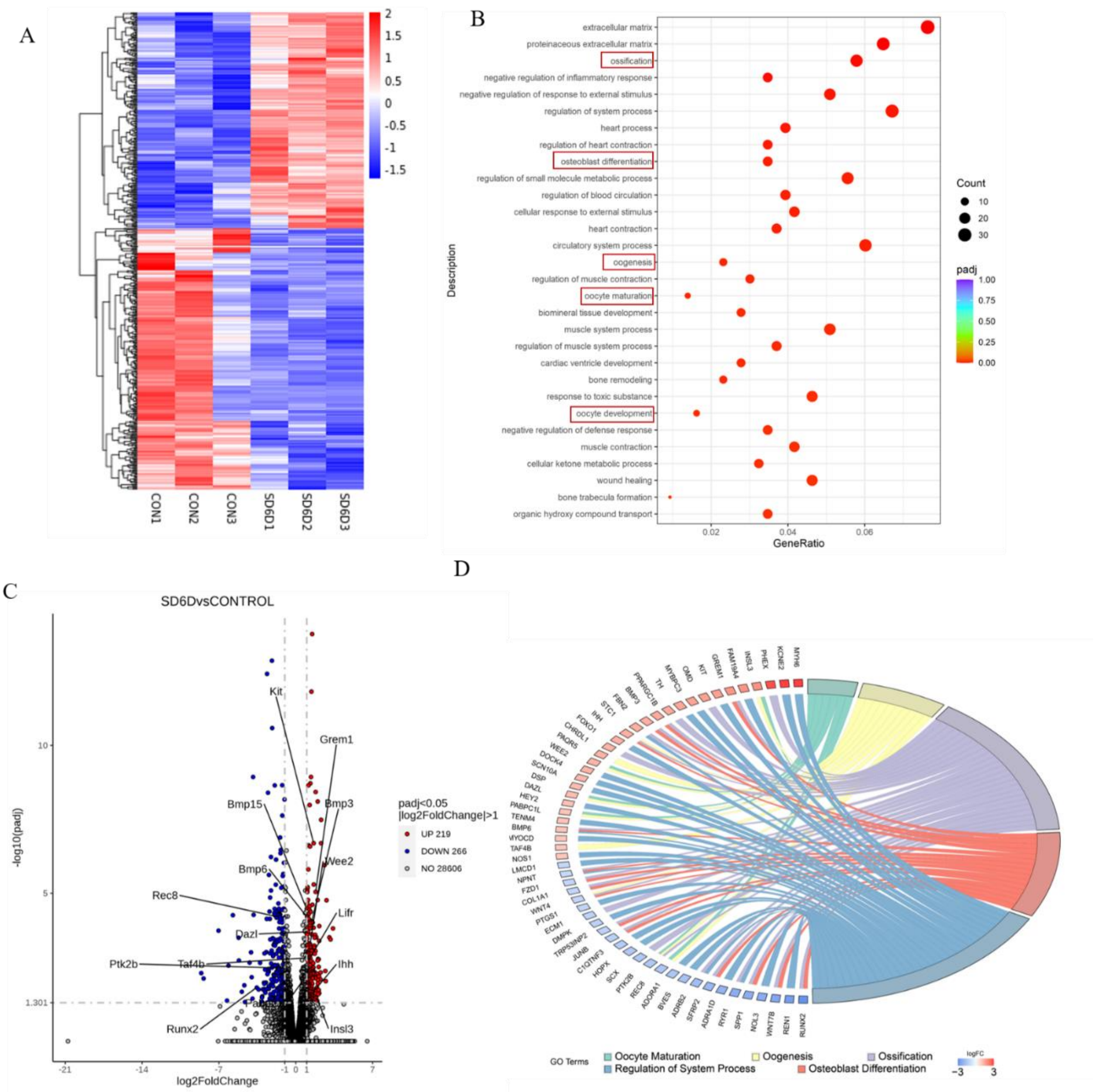

Figure S3. SD alters the mRNA expression profiles of mice ovary and genes associated with follicle development are enriched.

A) This heatmap illustrates the gene expression patterns in ovarian tissues of control and sleep-deprived mice. Samples were subjected to cluster analysis to group genes with similar expression patterns together. Red represents high expression, blue represents low expression, and the intensity of the color reflects the expression level.

B) The Gene Ontology (GO) plot displays a series of affected biological functions. The size of the circles reflects the importance of annotation or the number of genes included. The color of the circles represents the significance of the *p*-value in the statistical information.

C) Volcano map of Differentially Expressed Genes (DEGs) in ovarian tissue of mice after 6 days of SD (SD6D) compared to control (CON) mice. DEGs were identified by

mRNA sequencing and analyzed with *p*adj values of ≤ 0.05 and Log2 Fold Change values ≥ 1, with two vertical and one horizontal dashed gray lines distinguishing them. Significantly up-regulated and down-regulated DEGs are shown in red and blue, respectively. Genes with no significant changes are shown in gray.

D) The chord diagram illustrates the Gene Ontology (GO) terms associated with follicle development, including oocyte maturation, oogenesis, ossification, regulation of system process, and osteoblast differentiation. Different colors of clusters represent different pathways, with blue genes indicating down-regulation and red genes indicating up-regulation. CON, control; SD, sleep deprivation.

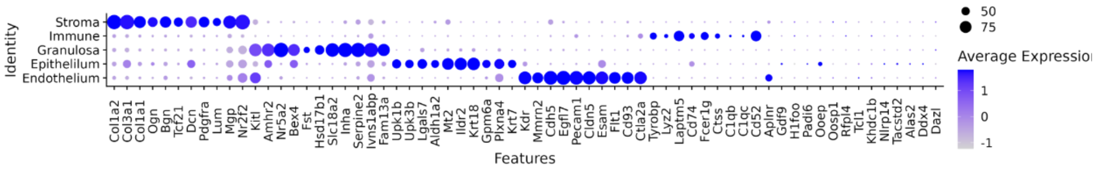

Figure S4 Bubble plot displaying single-cell sequencing marker expression of each cluster cell type, with darker shades of blue indicating higher expression levels and larger circles representing greater numbers of cells. The markers expressed by oocytes are within the red square.

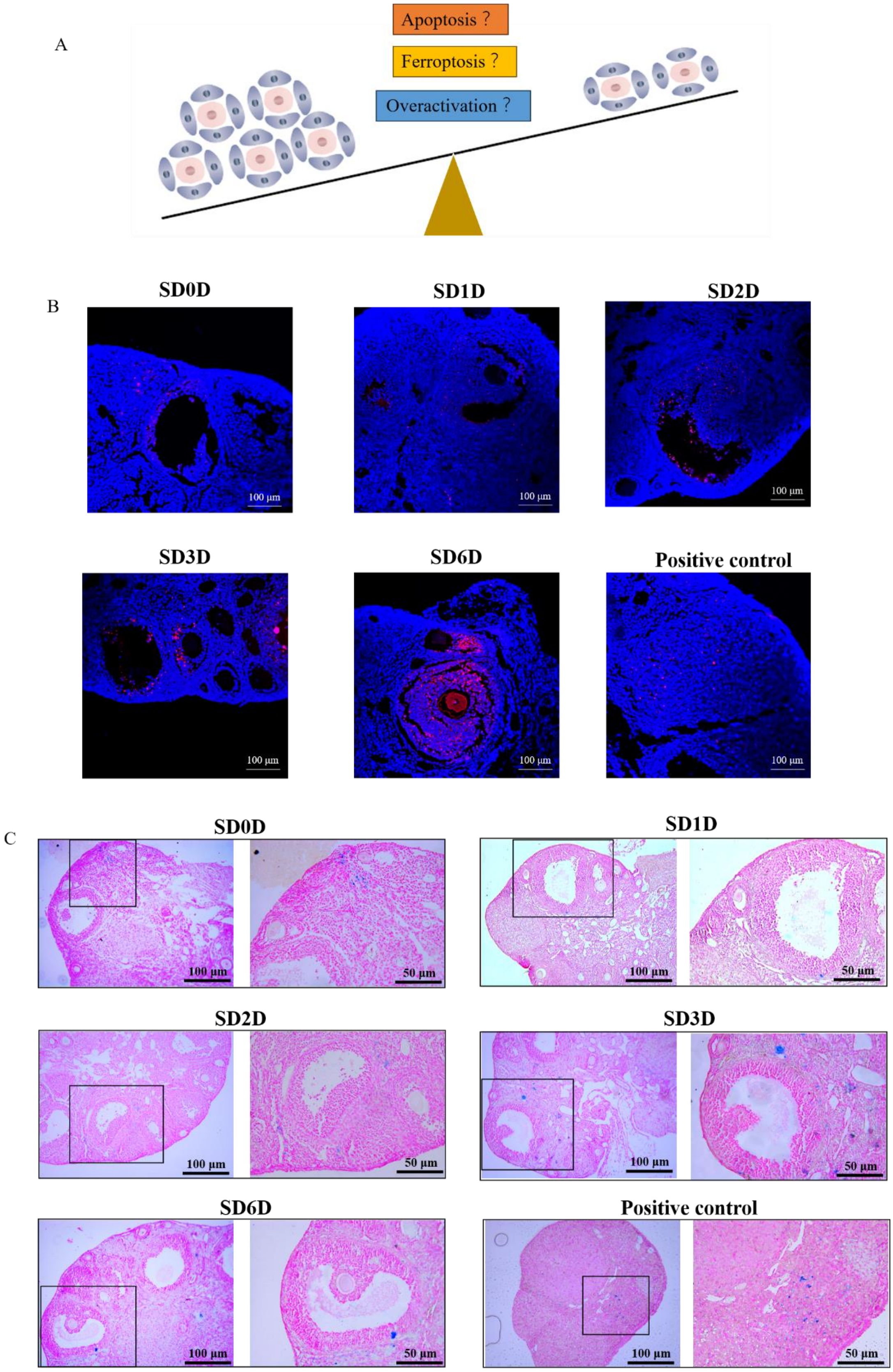

Figure S5. Possible mechanisms of SD-induced the loss of primordial follicles

A) Possible mechanism diagram by which SD affects the primordial follicles include

apoptosis, iron death, and overactivation of primordial follicles.

B) After SD, positive TUNEL staining were mainly observed in large antral developing follicles with red arrows, and no positive signals detected in primordial follicles. Scale bar, 100 μm.

C) Prussian blue staining revealed that positive signals in ovarian tissue after SD were mainly located in granulosa cells and ovarian stroma, with no positive signals found in primordial follicles. Scale bar, 100 and 50 μm. CON, control; SD, sleep deprivation.

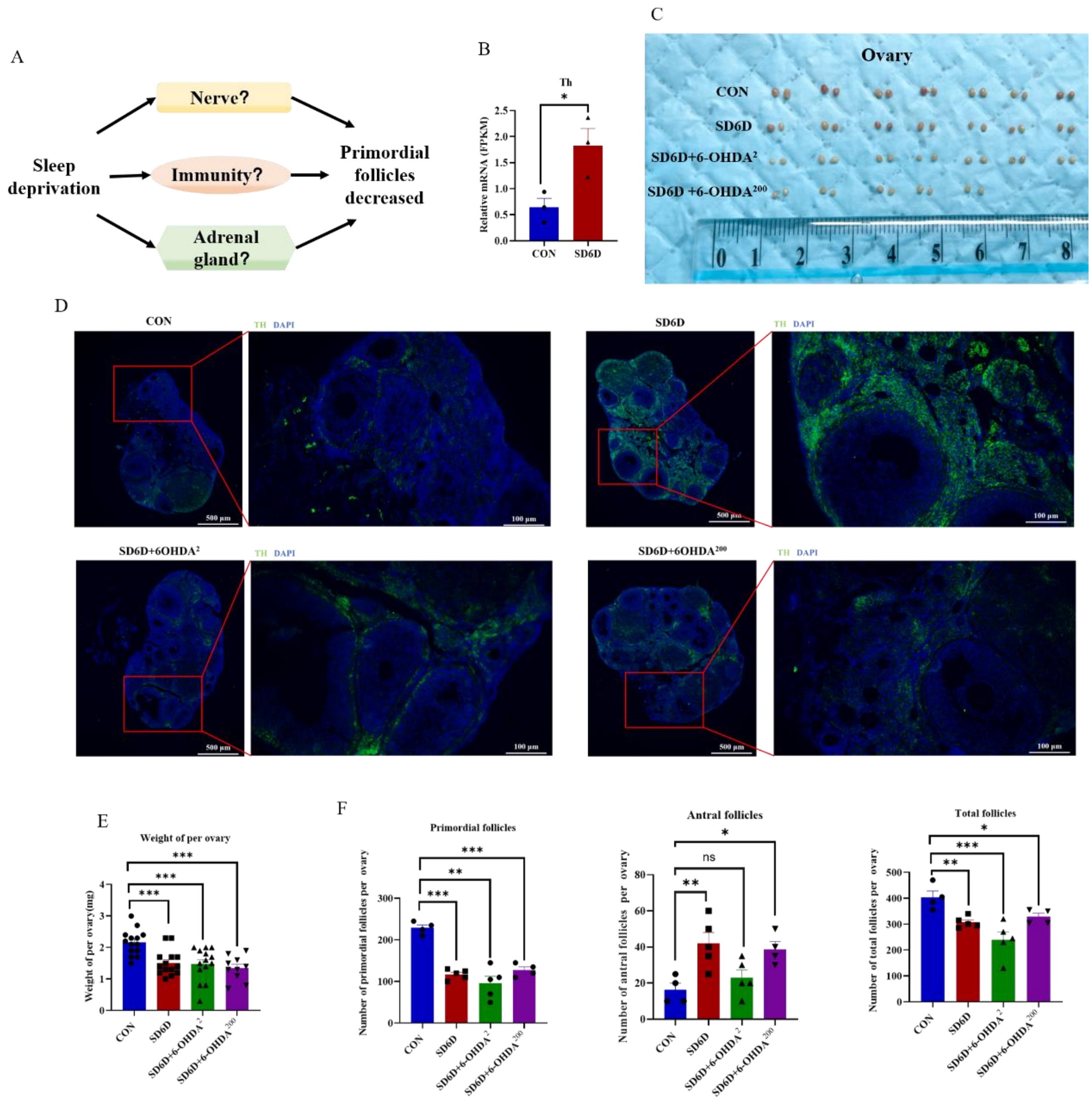

Figure S6. The loss of primordial follicles induced by SD is not dependent of sympathetic nerves.

A) The possible effects of SD transmitted to the ovaries and alter primordial follicles.

B) mRNA sequencing of ovaries after 6 days of SD compared to 0 days revealed significant upregulation of the sympathetic nerve marker Th (Tyrosine Hydroxylase). C, E) Mice were divided into four groups: CON group received no intervention, SD6D group underwent 6 days of SD, and SD6D+6-OHDA^{2} and SD6D+6-OHDA^{200} groups received different doses of 6-OHDA followed by 6 days of SD. All four groups of mice showed a significant decrease in ovarian weight after treatment (CON n = 14; SD6D n = 14; SD6D+6-OHDA^{2} n = 14; SD6D+6-OHDA^{200} n = 10). Statistical method: One-way ANOVA with LSD Test.

D) 6-OHDA is a drug used to ablate sympathetic nerves and was intraperitoneally

injected to mice at doses of 2mg/kg or 200mg/kg once. Representative images of Th staining in ovarian tissue of mice in the CON, SD6D, SD6D+6-OHDA (2mg/kg), and SD6D+6-OHDA (200mg/kg) groups. Scale bar, 500 and 100 μm

F) Number of primordial follicles, antral follicles, and total follicle count in mice from the CON, SD6D, SD6D+6-OHDA^{2} and SD6D+6-OHDA^{200} groups (CON n = 7; SD6D n = 10; SD6D+6-OHDA^{2} n = 8; SD6D+6-OHDA^{200} n = 10). Statistical methods: Primordial follicles: Independent-Samples Kruskal-Wallis Test; Antral follicles: One-way ANOVA with LSD Test; Total follicles: One-way ANOVA with LSD Test.

Data are presented as mean with SEM. In all panels, *$p< 0.05$, and ***$p< 0.001$, ns indicates not significant. CON, control; SD, sleep deprivation; SD6D+6-OHDA^{2}, Mice were administered 6-OHDA at a dose of 2 mg/kg and underwent 6 days of sleep deprivation; SD6D+6-OHDA^{200}, Mice were administered 6-OHDA at a dose of 200 mg/kg and underwent 6 days of sleep deprivation.

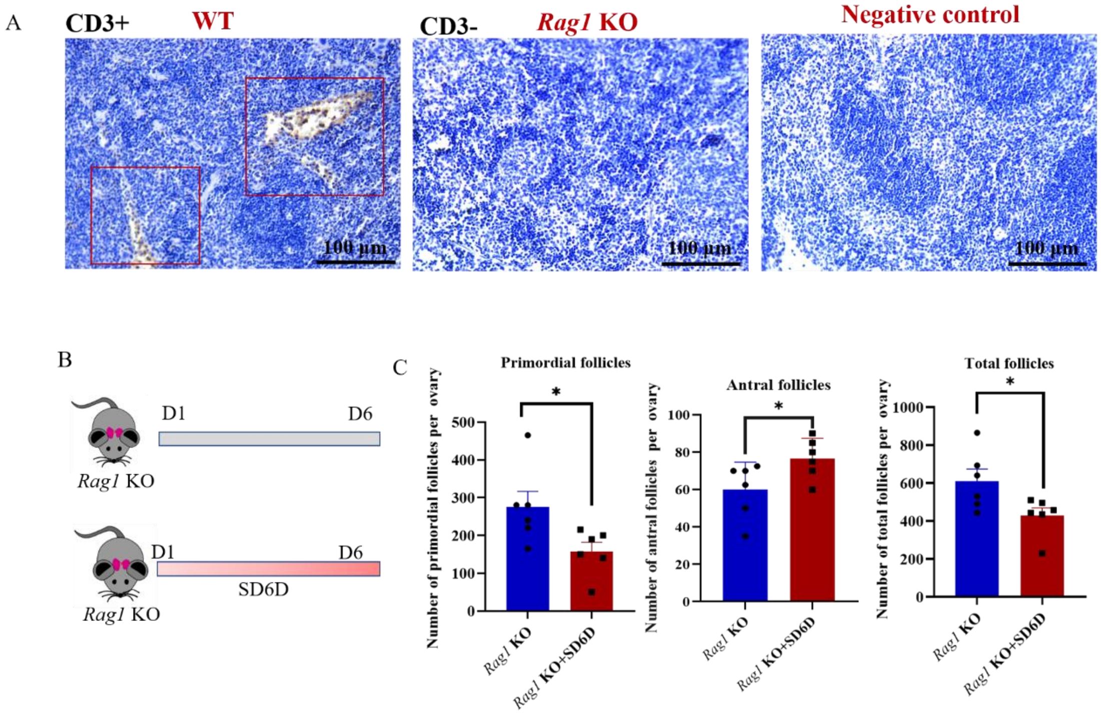

Figure S7. The loss of primordial follicles induced by SD is not dependent of immune attack.

A) *Rag1* KO mice, immunodeficient mice, with negative spleen CD3 staining. Scale bar, 100 μm.

B) Experimental design of SD for 6 days in *Rag1* KO mice.

C) Number of primordial follicles, antral follicles and total follicle count in *Rag1* KO mice after SD treatment (*Rag*1 KO n = 6; *Rag1* KO + SD n = 6). Statistical methods: Primordial follicles: Independent Samples Test; Antral follicles: Independent Samples Test; Total follicles: Mann-Whitney Test.

Data are presented as mean with SEM. In all panels, *$p< 0.05$. SD, sleep deprivation.

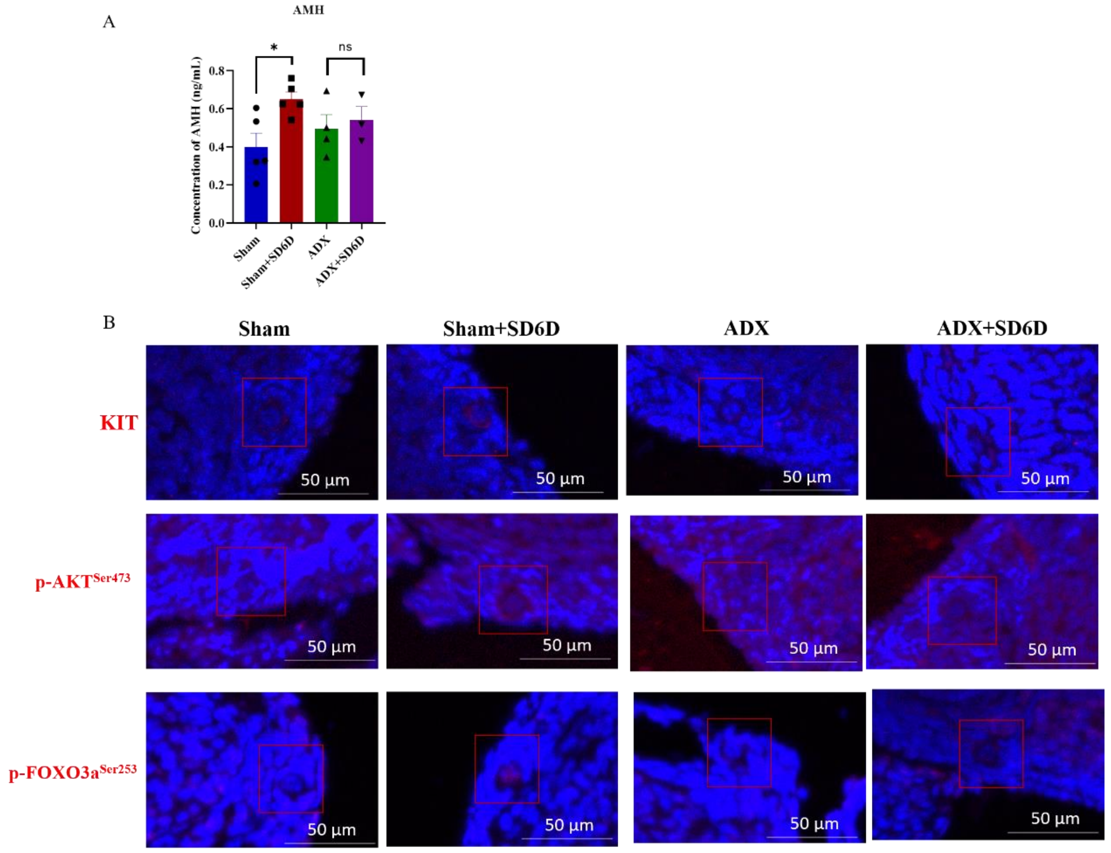

Figure S8. A) Changes in serum AMH levels after experimentation in four groups: Sham, Sham+SD, ADX, and ADX+SD mice (Sham, n = 5; Sham+SD6D n = 5; ADX n = 4; ADX+SD6D n = 4). Statistical method: One-way ANOVA with LSD Test.

B) Immunostaining of KIT, phosphorylated AKT and FOXO3a in ovarian primordial follicles. Immunostaining of ovarian tissue showing overexpression of KIT, p-AKT^{Ser473}, and p-FOXO3a^{Ser253} in primordial follicles in Sham+SD6D group compared with Sham group. and under-expression in ADX+SD6D group. After adrenalectomy followed by SD, this high expression was reversed. Scale bar, 50 µm.

Data are presented as mean with SEM. In all panels, *p< 0.05. SD, sleep deprivation; ADX, adrenalectomy.

.

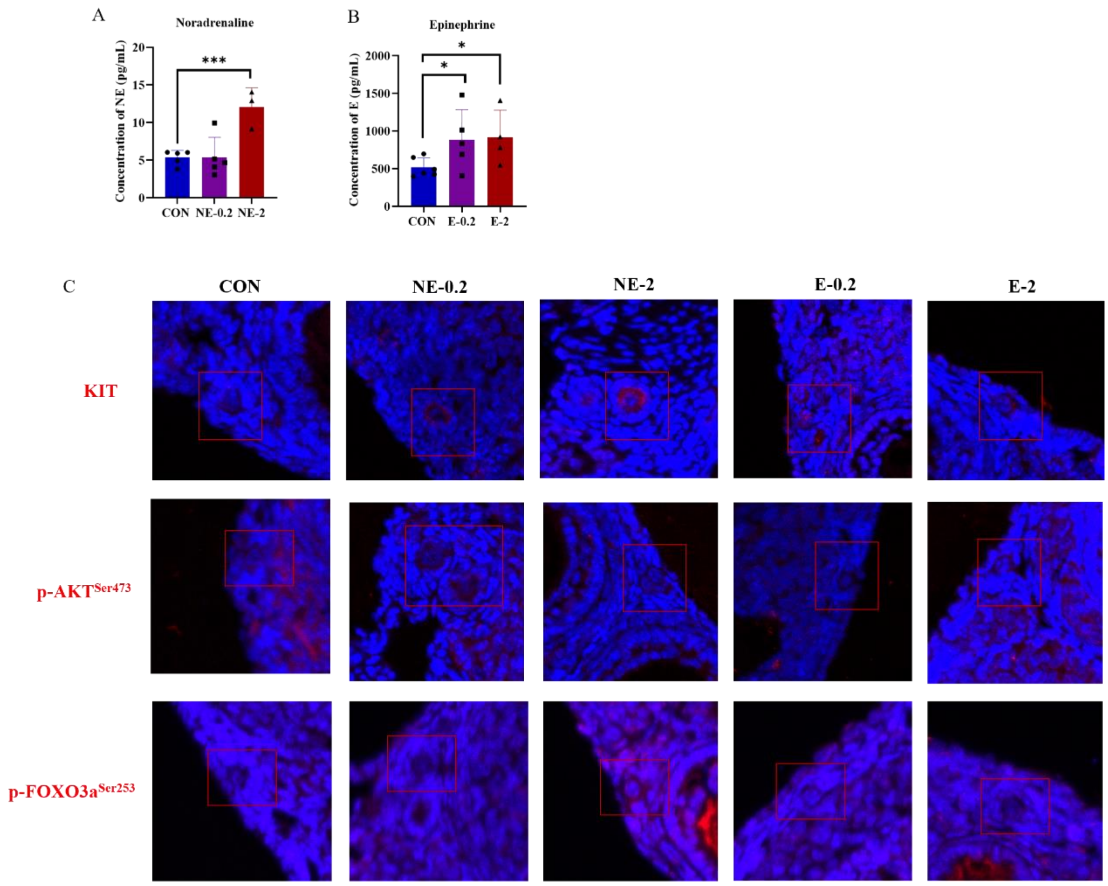

Figure S9. Immunostaining of KIT, phosphorylated AKT and FOXO3a in ovarian primordial follicles after treating with NE or E.

A) Peripheral serum NE concentration levels after injection of 0.2 mg/kg and 2 mg/kg NE in mice (CON n = 5, NE-0.2 n = 5, NE-2 n = 3). Statistical method: One-way ANOVA with LSD Test.
B) Peripheral serum E concentration levels after injection of 0.2 mg/kg and 2 mg/kg E in mice (CON n = 6, E-0.2 n = 5, E-2 n = 4). Statistical method: One-way ANOVA with LSD Test.

C) Immunofluorescence staining of the ovaries of 6-8 week-old wild-type mice indicates that ADRB2 is highly expressed in the granulosa cells of follicles at all stages.
D) Immunostaining of ovarian tissue showing overexpression of KIT, p-AKT^{Ser473}, and p-FOXO3a^{Ser253} in primordial follicles after treating with NE or E. Scale bar, 50 µm.

Data are presented as mean with SEM. In all panels, *p< 0.05, and ***p< 0.001, ns indicates not significant. CON, control; NE, noradrenaline; E, epinephrine.

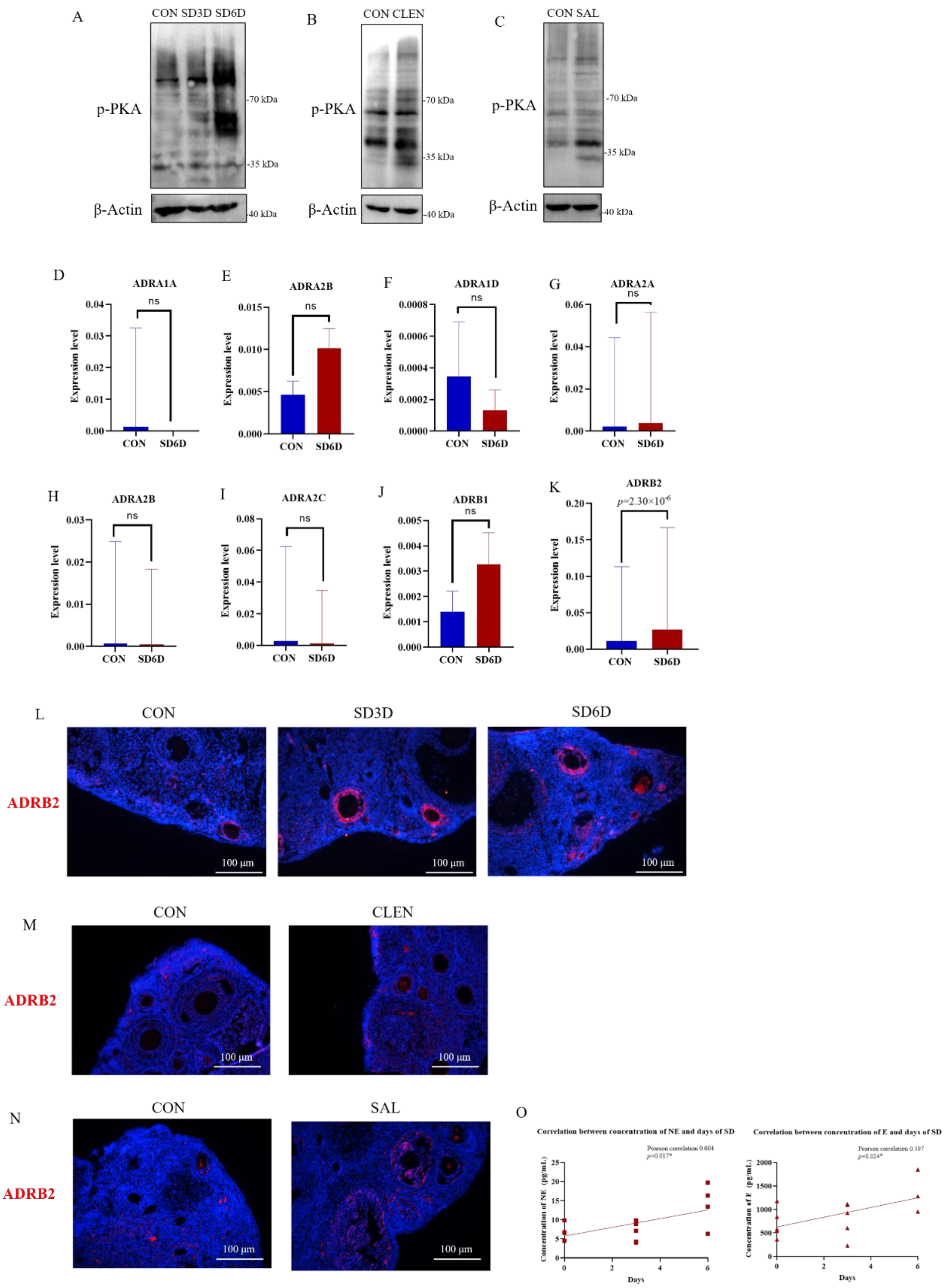

Figure S10. The impact of SD on adrenergic receptors in mouse ovarian granulosa Cells

A) The level of PKA phosphorylation downstream of ADRB2 in ovarian tissues of mice increases after sleep deprivation.

B, C) The level of PKA phosphorylation downstream of ADRB2 in ovarian tissues

increases after ADRB2 agonist treatment (CLEN or SAL) in mice.

D-K) Single cell RNA sequencing of SD mice ovaries showing expression levels of various types of adrenergic receptors in ovarian granulosa cells, with a notable upregulation of ADRB2.

L) Immunofluorescence analysis showing the high expression of ADRB2 in granulosa cells of ovarian tissue after SD. Scale bar, 100 μm.

M, N) Immunofluorescence analysis showing the upregulation of ADRB2 expression in the granulosa cells of ovarian tissue after ADRB2 agonist (CLEN, SAL). Scale bar, 100 μm.

O) Correlation analysis of serum NE and E concentrations with the duration of SD.

Data are presented as mean with SEM. In all panels, *p< 0.05. CON, control; SD, sleep deprivation; CLEN, Clenbuterol; SAL, Salbutamol.

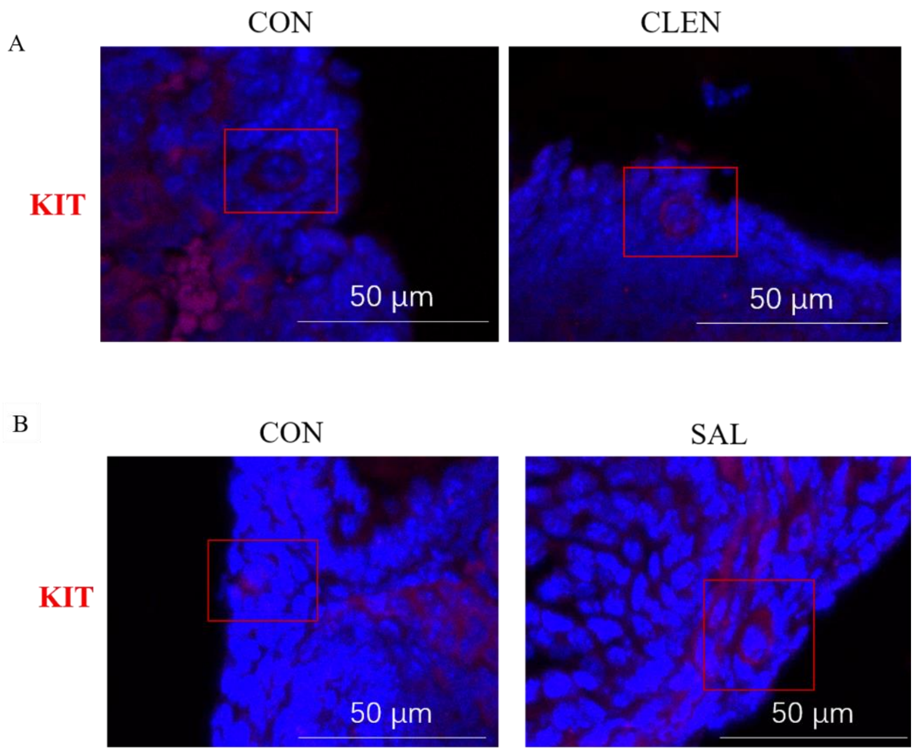

Figure S11. Immunostaining of KIT, phosphorylated AKT and FOXO3a in ovarian primordial follicles after treating with CLEN or SAL in mice ovaries.

A, B) Immunostaining of ovarian tissue showing overexpression of KIT in primordial follicles in CLEN and SAL groups compared with CON group. CON, control; CLEN, Clenbuterol; SAL, Salbutamol. Scale bar, 50 µm.

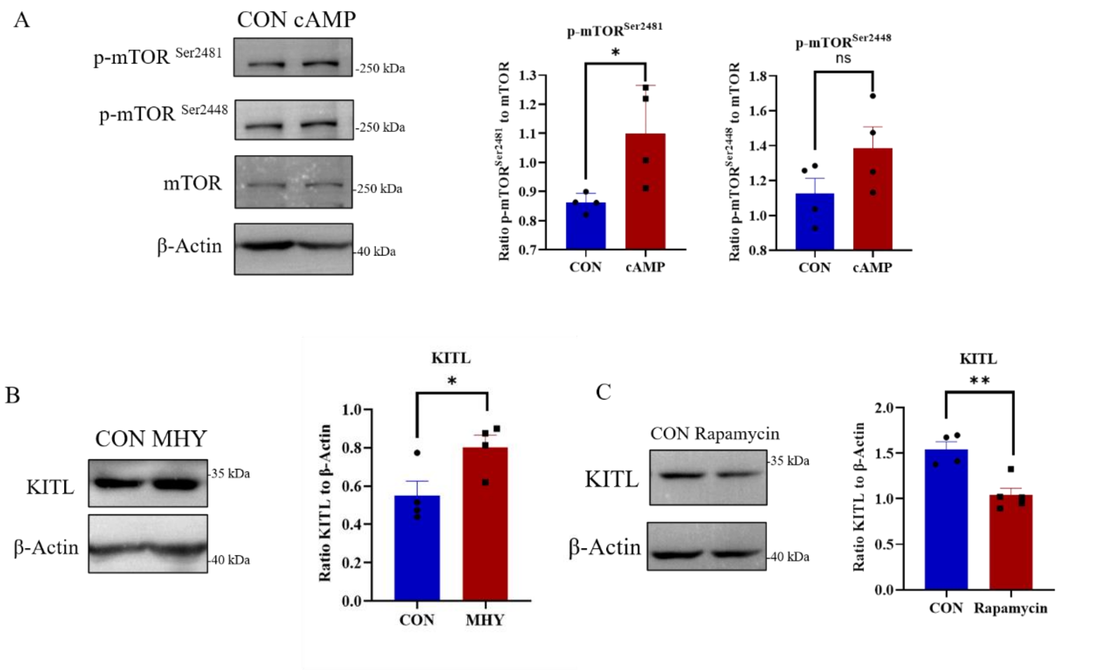

Figure S12. cAMP Activation of the mTOR Pathway Regulates KITL Expression in KGN Cells

A) Treatment with 20 μM cAMP significantly elevated the phosphorylation level of mTOR in KGN cells (CON n = 4, cAMP n = 4). Statistical method: p-mTOR ^{Ser2481}: Mann-Whitney Test; p-mTOR ^{Ser2448}: Independent Samples Test.

B) The expression level of KITL significantly increased after KGN cells were treated with 1 μM of the mTOR agonist MHY1485 for 24 hours (CON n = 4, MHY n = 4). Statistical method: Independent Samples Test.

C) The expression of KITL was significantly inhibited after KGN cells were treated with 0.1 μM of the mTOR antagonist Rapamycin for 24 hours (CON n = 4, Rapamycin n = 5). Statistical method: Independent Samples Test.

Data are presented as mean with SEM. In all panels, *p< 0.05; **p< 0.01. CON, control; MHY, MHY1485.

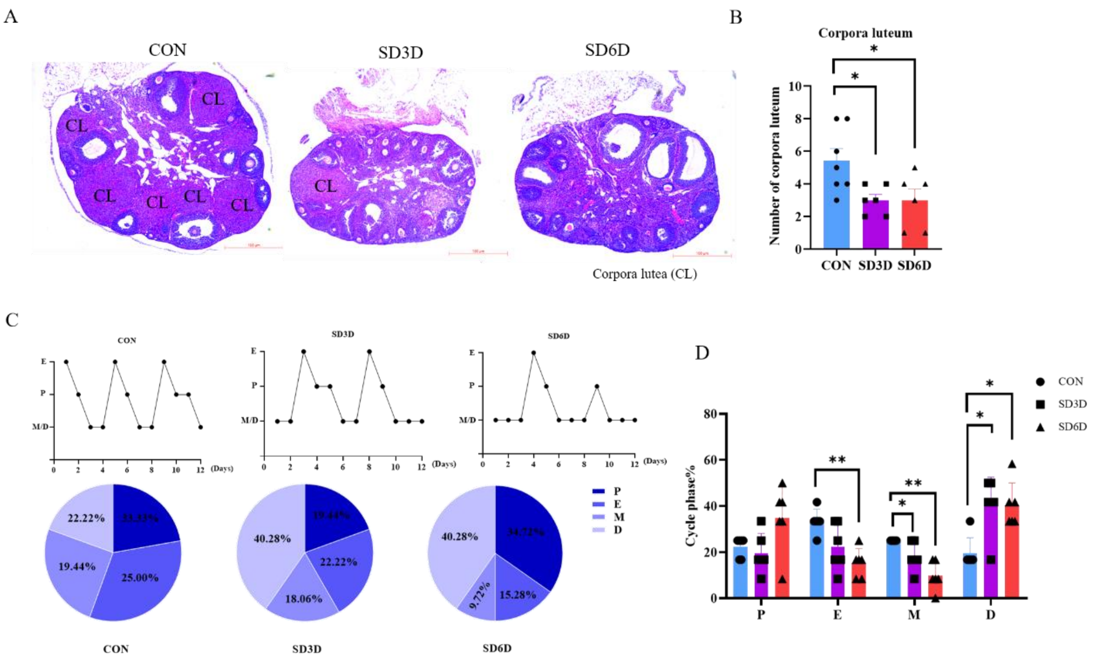

Figure S13. SD significantly reduced the number of corpus luteum and disturbed of estrous cycle

A, B) SD significantly reduced the number of corpus luteum in the ovaries of mice (CON n = 6; SD3D n = 6; SD6D n = 6). Scale bar, 200 μm. Statistical method: One-way ANOVA with Tamhane's T2 test.

C) Representative record of estrous cycle in three groups of mice

D) Statistical analysis of each estrous cycle in three groups of mice (CON n = 6; SD3D n = 6; SD6D n = 6). Statistical method: P, D: Independent-Samples Kruskal-Wallis Test; E: One-way ANOVA with LSD test; M: One-way ANOVA with Tamhane's T2 test.

Data are presented as mean with SEM. **p*< 0.05, ***p*< 0.01. CON, control; SD, sleep deprivation; P, proestrus; E, estrus; M, metestrus; D, diestrus.

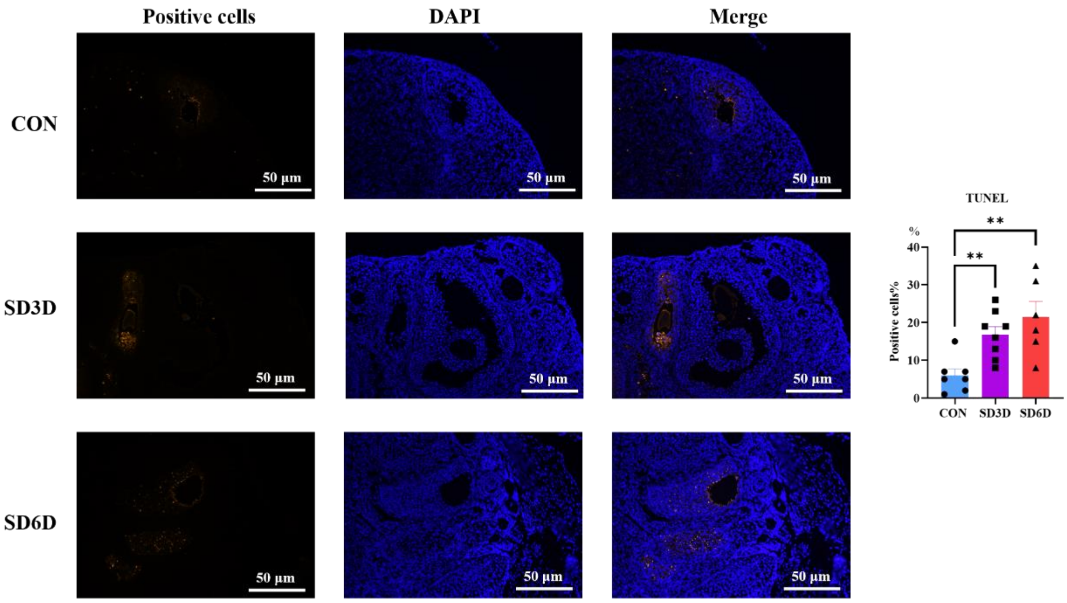

Figure S14. Sleep deprivation significantly increases apoptosis in granulosa cells of antral follicles in the ovary.

After sleep deprivation, the proportion of TUNEL-positive granulosa cells in antral follicles of mice ovaries significantly increased (CON n = 6; SD3D n = 6; SD6D n = 6). Statistical method: One-way ANOVA with LSD test. Scale bar, 50 μm.

Data are presented as mean with SEM. ***p*< 0.01. CON, control; SD, sleep deprivation.

Source Data of Western blot in Supplementary Figures.

Related to Figure 3 PI3K and p-PI3K

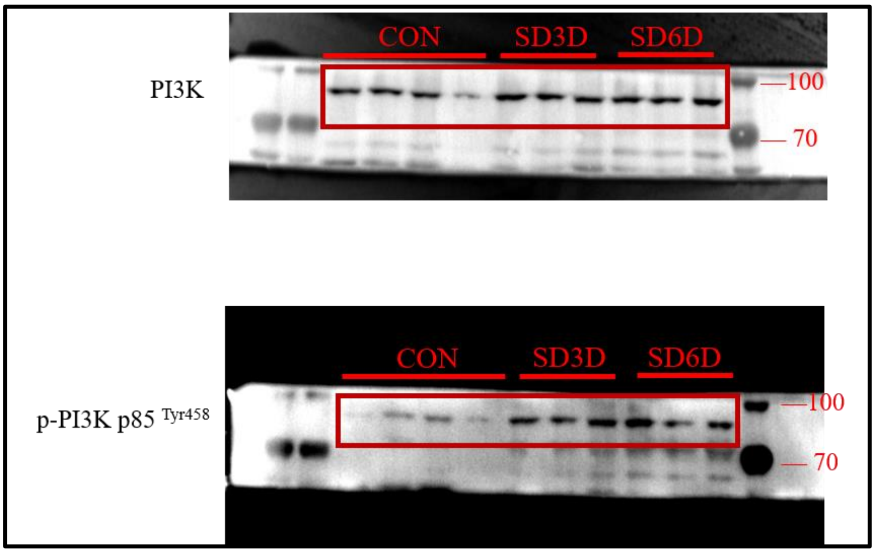

Related to Figure 3 AKT and p-AKT

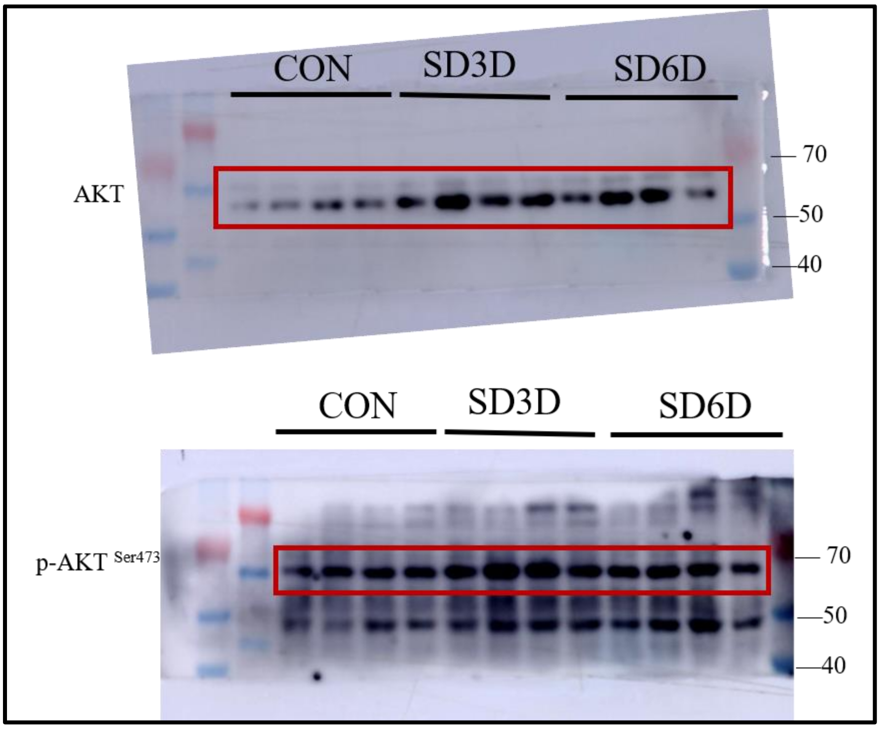

Related to Figure 3 mTOR and p-mTOR

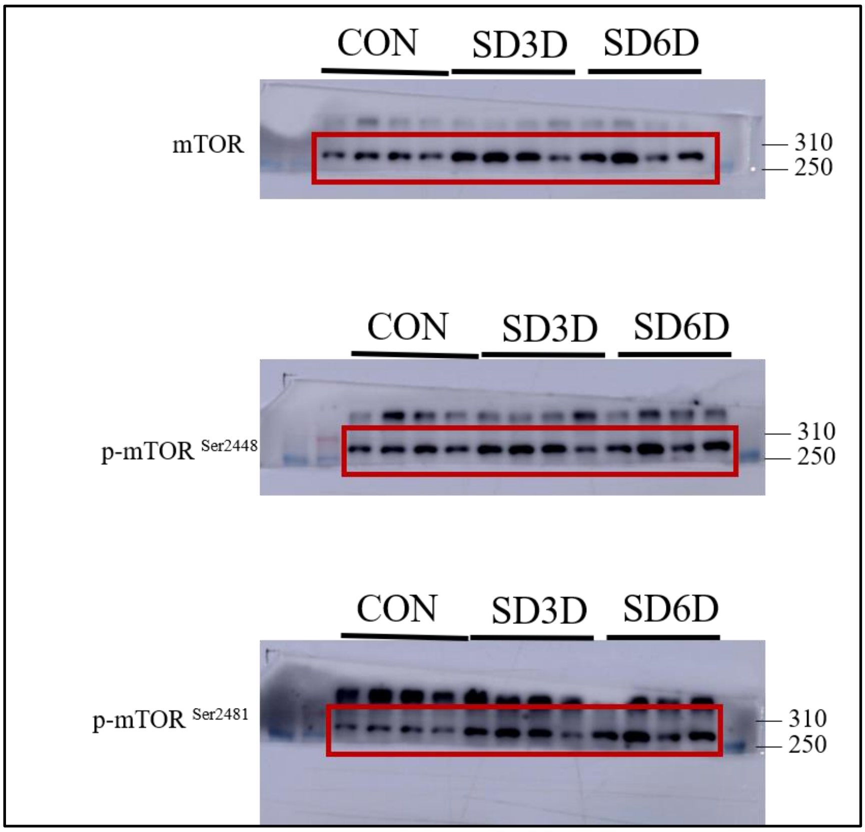

Supplement: Supplementary file 1 — Supporting Information [file ADVS-11-2402393-s001.pdf]
